# β-Secretase (BACE1) inhibition causes retinal pathology by vascular dysregulation and accumulation of age pigment

**DOI:** 10.1002/emmm.201101084

**Published:** 2012-08-20

**Authors:** Jun Cai, Xiaoping Qi, Norbert Kociok, Sergej Skosyrski, Alonso Emilio, Qing Ruan, Song Han, Li Liu, Zhijuan Chen, Catherine Bowes Rickman, Todd Golde, Maria B Grant, Paul Saftig, Lutgarde Serneels, Bart de Strooper, Antonia M Joussen, Michael E Boulton

**Affiliations:** 1 Department of Anatomy & Cell Biology, University of FloridaGainesville, FL, USA; 2 Department of Ophthalmology, Charité Universitätsmedizin BerlinBerlin, Germany; 3 Department of Surgery, University of FloridaGainesville, FL, USA; 4 Department of Pharmacology & Therapeutics, University of FloridaGainesville, FL, USA; 5 Department of Ophthalmology & of Cell Biology, Duke University Medical CenterDurham, NC, USA; 6 Department of Neuroscience, Center for Translational Research in Neurodegenerative Disease, University of FloridaGainesville, FL, USA; 7 Biochemical Institute, Christian-Albrecht's UniversityKiel, Germany; 8 Center for Human Genetics and Leuven Institute for Neurodegenerative Diseases (LIND)KU Leuven, Leuven, Belgium; 9 VIB Center for the Biology of Disease, Vlaams Instituut voor BiotechnologieLeuven, Belgium

**Keywords:** angiogenesis, β-secretase, lipofuscin, retina, retinal pigment epithelium

## Abstract

β-Secretase (BACE1) is a major drug target for combating Alzheimer's disease (AD). Here we show that BACE1^−/−^ mice develop significant retinal pathology including retinal thinning, apoptosis, reduced retinal vascular density and an increase in the age pigment, lipofuscin. BACE1 expression is highest in the neural retina while BACE2 was greatest in the retinal pigment epithelium (RPE)/choroid. Pigment epithelial-derived factor, a known regulator of γ-secretase, inhibits vascular endothelial growth factor (VEGF)-induced *in vitro* and *in vivo* angiogenesis and this is abolished by BACE1 inhibition. Moreover, intravitreal administration of BACE1 inhibitor or BACE1 small interfering RNA (siRNA) increases choroidal neovascularization in mice. BACE1 induces ectodomain shedding of vascular endothelial growth factor receptor 1 (VEGFR1) which is a prerequisite for γ-secretase release of a 100 kDa intracellular domain. The increase in lipofuscin following BACE1 inhibition and RNAI knockdown is associated with lysosomal perturbations. Taken together, our data show that BACE1 plays a critical role in retinal homeostasis and that the use of BACE inhibitors for AD should be viewed with extreme caution as they could lead to retinal pathology and exacerbate conditions such as age-related macular degeneration.

## INTRODUCTION

β-Secretase (BACE1) is an aspartyl protease that catalyses the rate limiting step in the production of the β-amyloid protein (Aβ) by cleaving the amyloid precursor protein (APP) in its ectodomain (De Strooper et al, 2010; Vassar et al, 2009). BACE1 provides a promising therapeutic target for Alzheimer's disease (AD) and numerous BACE1 inhibitors have been designed, of which several have gone into clinical trials (Klaver et al, 2010; Vassar et al, 2009; Woo et al, 2011). However, the clinical development of LY2811376 (Eli Lilly) was recently stopped due in part to retinal pathology in rats (May et al, 2011). The investigators concluded that this was an off target effect of their compound rather than the direct effect of BACE inhibition. As BACE1 cleaves a variety of substrates in addition to APP (*e.g.* neuregulin, β-subunits of the voltage-gated sodium channels, interleukin-1 receptor 2, low-density lipoprotein (LDL) receptor-related protein) (Klaver et al, 2010; Vassar et al, 2009; Woo et al, 2011) it is likely to have other crucial physiological consequences. Therefore, it is critically important to monitor carefully the potential side effects of BACE inhibition.

## RESULTS

**Figure 1:**
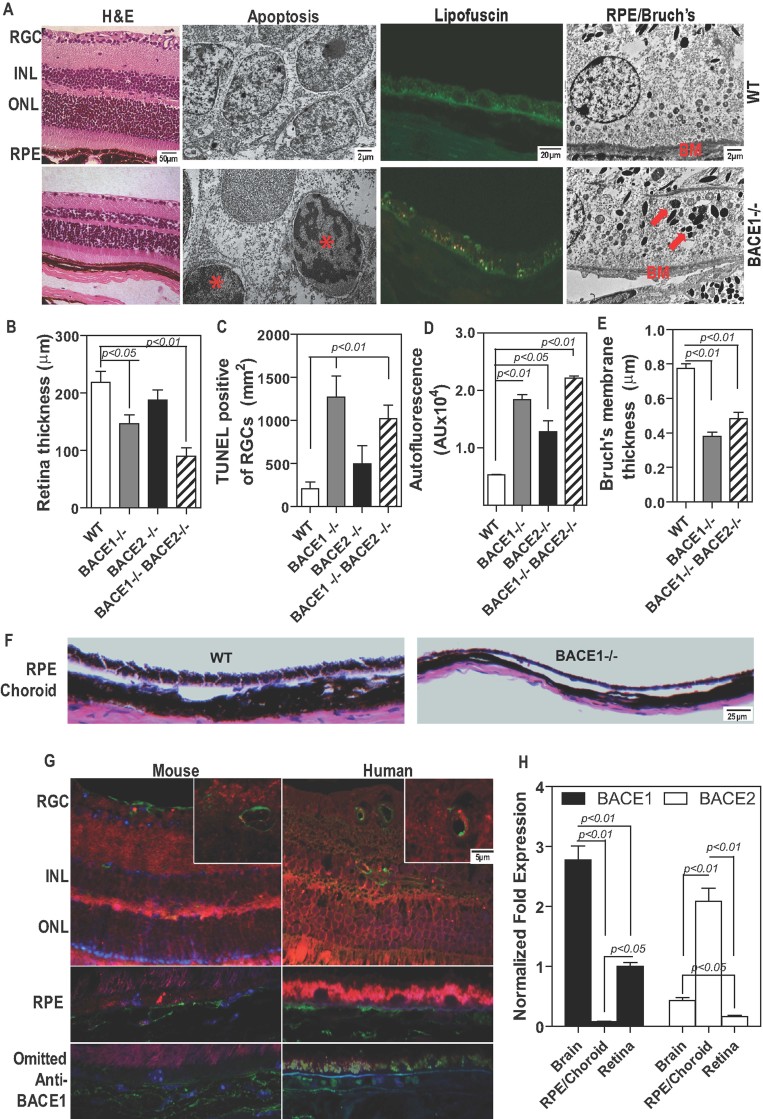
Morphological analysis of BACE1 knockout mice and BACE expression in the normal retina **A.** Representative haematoxylin/eosin staining of retinas from 4-month-old wild-type (WT) and BACE1^−/−^ mice. Knockout animals demonstrated shrunken retinal ganglion cells (RGCs), depopulation in the ganglion cell layer, thinning of the retina and inner nuclear layer (INL) and outer nuclear layer (ONL) degeneration. Eyes of wild-type mice (WT) were histologically normal. Transmission electron micrographs taken from the inner layer of the BACE1^−/−^ retina showed shrunken RGCs and neuronal cells with hyperchromatic and condensed nuclear chromatin, indicative of neuronal apoptosis (*), while cells from WT showed normal ultrastructure. Confocal micrographs of the RPE revealed an increase in lipofuscin granules in BACE1^−/−^ animals compared to WT. Electron micrographs of BACE1^−/−^ RPE/choroid appeared to contain increased lipofuscin and thinning in Bruch's membrane (BM) compared to WT.**B–E.** Quantitative analysis showing differences in (**B**) retinal thickness, (**C**) apoptosis, (**D**) lipofuscin autofluorescence and (**E**) Bruch's membrane thickness in BACE1^−/−^, BACE2^−/−^ and BACE1^−/−^ BACE2^−/−^ mice compared to WT (mean ± SEM, *n* = 5).**F.** Representative haematoxylin/eosin staining of the RPE showing marked thinning of the RPE layer in BACE1^−/−^ mice compared to wild type.**G.** Detection of BACE1 expression in the retina of wild-type mouse (4-month-old) and human eyes (39-year-old donor). Paraffin sections were immunostained using an antibody against BACE1 (red) and dual stained with agglutinin–FITC (green) to visualize the vasculature. BACE1 expression was observed in all layers of the retina, however, strongest staining was localized to the inner and outer plexiform layers and the retinal vasculature in both mouse and human. BACE1 was weakly localized in both RPE and the choroid.**H.** mRNA levels of BACE1 and BACE2 in mouse brain, RPE choroid and retina from 4-month-old animals were analyzed by quantitative PCR. BACE expression was plotted using fold values with housekeeping gene GAPDH. The data was represented as mean ± SEM (*n* = 6).

β-Secretase is expressed in the rodent retina (Xiong et al, 2007) and deposition of Aβ is observed in aged animals (Anderson et al, 2004; Ding et al, 2011; Yoshida et al, 2005). We therefore explored whether BACE1 knockout could result in retinal pathology. In BACE1^−/−^ knockout animals the neural retina shows distinct thinning (Fig 1A, Supporting Information Fig S1A) which was reduced by approximately 50% in the inner nuclear layer (INL) and 35% in the outer nuclear layer (ONL) of the retina compared to wild-type (WT) littermates (Fig 1B). BACE1^−/−^ animals demonstrated a reduction in photopic electroretinography (ERG; the cone photoreceptor response under well-lit conditions allowing colour perception) (Supporting Information Fig S1B) but no change in scotopic ERG (the rod photoreceptor response under low light conditions) (data not shown). Shrunken and atrophic retinal ganglion cells (RGCs), which were hyperchromatic were observed in the ganglion cell layer. This was confirmed by transmission electron microscopy which showed typical neuronal apoptosis and the TdT-mediated dUTP nick end labelling (TUNEL) assay which displayed a significant increase in apoptotic nuclei compared to WT animals (Fig 1A and C, Supporting Information Fig S1C). A marked increase in the age pigment lipofuscin is observed in BACE1^−/−^ mice (Fig 1A and D, Supporting Information Fig S1D and E) and areas of retinal pigment epithelium (RPE) thinning and atrophy are observed (Fig 1F) which are strongly associated with retinal degenerative diseases (Sparrow & Boulton, 2005). The areas of atrophy were always associated with elevated lipofuscin. The underlying Bruch's membrane of BACE1^−/−^ shows marked reduction in thickness compared to WT mice (Fig 1A and E). By contrast, these changes are not observed in WT animals (Fig 1A–F). Overall, BACE1^−/−^ retinal pathology did not change after 4 months of age. We observe a different, and milder, retinal phenotype in BACE2^−/−^ mice (Fig 1B–E, Supporting Information Fig S2) even though BACE2 shares 68% homology with BACE1 (Solans et al, 2000). Overall the neural retina appears relatively normal although occasional foci of neural retinal hyperplasia are observed (Supporting Information Fig S2A). BACE2^−/−^ mice exhibit a highly disrupted choroid (Supporting Information Fig S2). BACE2^−/−^ animals exhibit a 1.5-fold increase in lipofuscin autofluorescence but this is significantly less than the 2.5-fold increase observed in BACE1^−/−^ mice (Fig 1D, Supporting Information Fig S2). Autofluorescence fundus images of BACE1^−/−^ mice exhibited a white ‘shadow’ around the main vessels suggestive of inflammation while in BACE2^−/−^ mice there were white dots concentrated at the optic nerve indicating focal areas of lipofuscin hyperfluorescence (Supporting Information Fig S2D). BACE1^−/−^BACE2^−/−^ double knockout mice exhibit a retinal phenotype similar to BACE1^−/−^ mice with the surprising observation that the choroidal defect seen in the BACE2^−/−^ mice is absent (Fig 1B–E, Supporting Information Fig S2). This suggests that the ratio of BACE1 to BACE 2 may be critical in regulating the choroidal vasculature. Expression of BACE1 is highest in the neural retina of both normal mouse and human specimens, while BACE2 expression is highest in the RPE/choroid and lowest in the neural retina (Fig 1G, Supporting Information Fig S3A). This was confirmed by qRT-PCR which showed high levels of expression of BACE1 in the mouse neural retina and greatly reduced, but significant, expression in the RPE/choroid (Fig 1H). BACE1 messenger RNA (mRNA) expression in the mouse neural retina is less than 50% of that in the brain. As expected, staining for BACE1 and BACE2 is absent from their respective knockouts while the complementary BACE is expressed at levels consistent with WT controls (Supporting Information Fig S3B).

**Figure 2:**
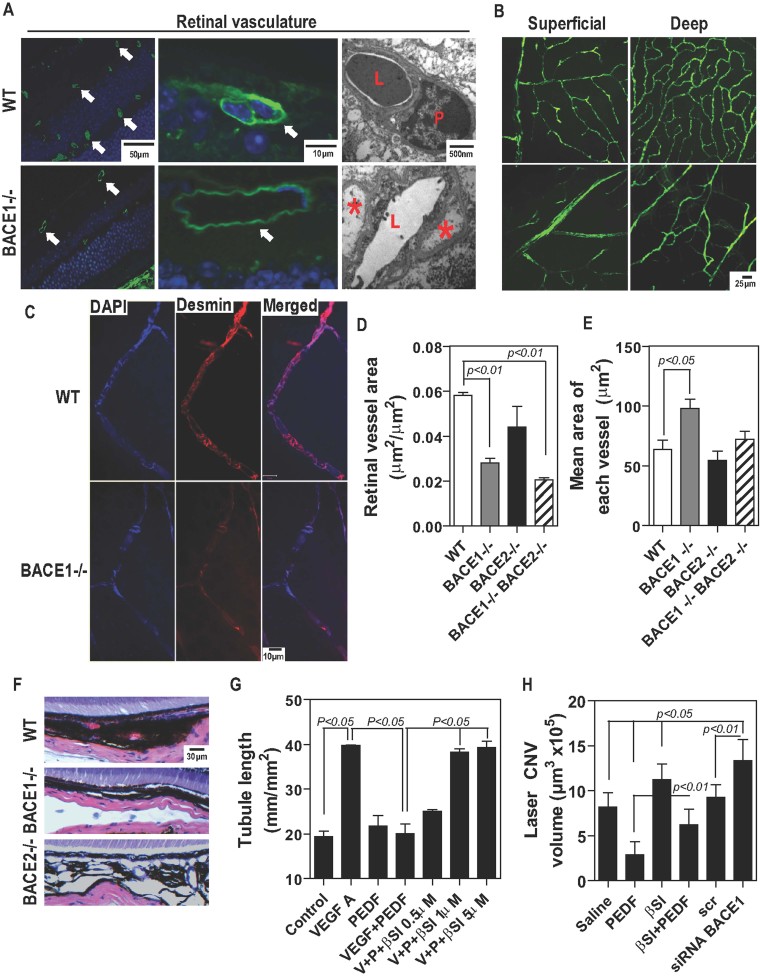
Changes in the retinal microvasculature of BACE knockout mice and the effect of BACE1 inhibition on *in vitro* and *in vivo* angiogenesis Animals were 4 months old and age-matched with WT. BACE1^−/−^ retinal sections stained with agglutinin–FITC to visualize the retinal vasculature showed decreased retinal capillaries (arrows) compared to the normal organization and distribution of retinal capillaries observed in WT mice. Higher magnification images showing vascular changes and the presence of atypical, enlarged capillaries (lack of the DAPI-blue nuclear stain) in BACE1^−/−^ mice compared to WT. Electron micrograph of retinal vessels from WT mice revealed normal vessel filled lumens (L) with blood cells, encircled by basement membrane and adjacent pericytes while in vessels of BACE1^−/−^ mice there was loss of pericytes (*) and abnormal vascular endothelial cells.Retinal flatmounts from animals that had been perfused with dextran-FITC confirming loss of vessels in both the superficial and deep vascular plexus in BACE1^−/−^ mice compared to wild type.Detection of pericytes in the retina of wild-type and BACE1^−/−^ mice. Flat mount retinas were immunostained using an antibody against desmin (red) and dual stained with DAPI (blue). Desmin expression is greatly reduced in BACE1^−/−^ mice compared to wild type.Quantitative analysis of the retinal vessel area per unit area of the retina revealed a significant decreases in knockout mice compared with WT (mean ± SEM, *n* = 6).Quantitative analysis of the mean retinal area of retinal vessels revealed a significant increases in knockout mice compared with WT (mean ± SEM, *n* = 6).Representative haematoxylin/eosin staining of the RPE and choroid showing a highly disorganized choroid in BACE2^−/−^ mice.Quantification of length of tubule formation (mm/mm^2^) of microvascular endothelial cells in the Matrigel™. Cells were pretreated with VEGF (100 ng/ml) and/or PEDF (100 ng/ml), in the presence or the absence of 0.5, 1 or 5 µM β-secretase inhibitor IV (β-SI). Results are mean ± SEM of at least three independent experiments.Quantitative assessment of the volume of the vascular lesions induced by laser-damage to eyes of mice receiving intravitreal injection of either PEDF, BACE1 inhibitor (βSI), a combination of the two, BACE1 siRNA, scrambled siRNA (scr) or saline vehicle control. Animals were sacrificed 14 days postlaser injury and RPE choroidal flat mounts were stained with a vascular specific marker, agglutinin-TRITC conjugate to visualize the CNV lesions and volume was determined by confocal microscopy (mean ± SEM, *n* = 6).

Previously, we reported that γ-secretase is a critical regulator of ocular angiogenesis (Boulton et al, 2008a, b; Cai et al, 2006, 2011a). We therefore assessed the influence of BACE1 knockout on retinal vasculature changes. Staining of the retinal vasculature with agglutinin-fluorescein isothiocyanate (FITC) demonstrates decreased retinal capillary density in both the superficial and deep retinal plexus for BACE1^−/−^ compared to WT controls (Fig 2A) and this was confirmed in retinal flat mounts (Fig 2B). Electron microscopy provides evidence of pericyte loss (Fig 2A) which was confirmed by a reduction in desmin immunostaining for pericytes in retinal flat mounts (Fig 2C). Furthermore, retinal vessels with reduced or atypical endothelial cells were also apparent (Supporting Information Fig S2A). Quantitation of vascular area indicates that this is reduced by about 40% in both BACE1^−/−^ and BACE1^−/−^ BACE2^−/−^ animals but not significantly altered in BACE2^−/−^ mice, compared to WT control (Fig 2D) and this was confirmed by immunohistochemistry (Supporting Information Fig S4B). Furthermore, the average area of individual superficial vessels was significantly increased in BACE1^−/−^ mice when compared to WT (Fig 2E). The choroidal vasculature appeared normal in BACE1^−/−^ animals but was severely disrupted in BACE2^−/−^ animals compared to control (Fig 2F). Pigment epithelial-derived factor (PEDF; a potent anti-angiogenic factor Tombran-Tink, 2005), promotes γ-secretase-dependent cleavage of vascular endothelial growth factor receptor 1 (VEGFR1) and negatively regulates angiogenesis (Cai et al, 2006). We assessed the effect of PEDF in combination with BACE1 inhibition on *in vitro* angiogenesis (Fig 2G, Supporting Information Fig S4C and D). As previously reported (Cai et al, 2006), PEDF abolishes vascular endothelial growth factor (VEGF)-induced tubule formation, proliferation and migration in *in vitro* angiogenesis models. However, addition of BACE1 inhibitor blocks the effects of PEDF in a dose-dependent manner (Fig 2G, Supporting Information Fig S4C and D). The inhibitory effect of PEDF on VEGF-stimulated tubule formation and endothelial cell proliferation is counteracted at 1 µM BACE inhibitor but 5 µM is required to block migration. The effect of BACE1 inhibition on *in vitro* angiogenesis is recapitulated *in vivo* using the laser-induced model of choroidal neovascularization (CNV) (Fig 2H). PEDF inhibited CNV and this inhibition is blocked by BACE1 inhibition. Furthermore, inhibition of BACE1 alone increases the level of CNV compared to untreated control. Western blot analysis confirms that PEDF stimulates a time-dependent increase in BACE1, six-fold by 24 h for endothelial cells but not for RPE cells (Supporting Information Fig S4E).

**Figure 3:**
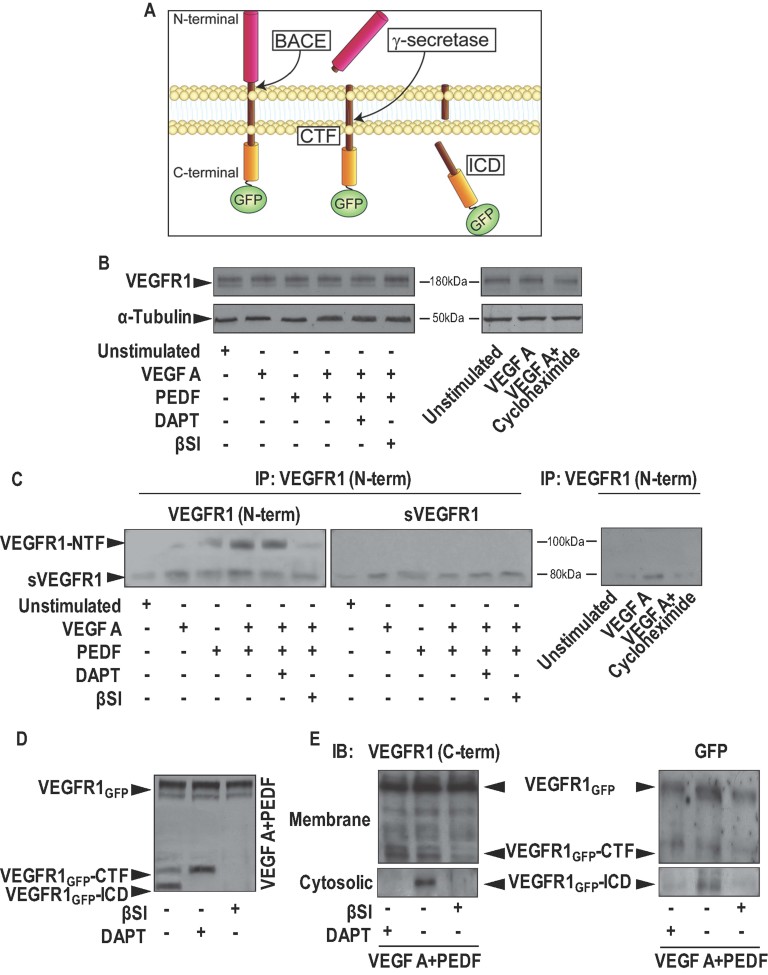
BACE1 is critical for ectodomain cleavage of VEGFR1 Porcine aortic endothelial cells (PAECs) were stably transfected with wild-type pVEGFR1-GFP wild-type and treated with VEGF (100 ng/ml), PEDF (100 ng/ml), PEDF + VEGF for 24 h, with or without BACE1 inhibitor (β-SI; 10 µM) or γ-secretase inhibitor DAPT (10 µM). Representative western blots from 4 independent experiments. Diagram depicting the sequential stages of VEGFR1 cleavage and the fragments generated. CTF = C-terminal fragment, ICD = intracellular domain.Western blot of the lysates for VEGFR1 demonstrated no major change in total VEGFR1 expression except in the presence of cycloheximide.Lysates were immunoprecipitated (IP) with an antibody against the VEGFR1 N-terminal domain and immunoblotted for either the N-terminal domain of VEGFR1 or native sVEGFR1. PEDF and PEDF + VEGF treatments resulted in the appearance of a VEGFR1-NTF fragment which was blocked by BACE1 inhibition. sVEGFR1 was not affected by BACE1 inhibition. The effect of cycloheximide on sVEGFR1 levels in the culture medium confirmed that the sVEGFR1 is translationally regulated and therefore not derived from ectodomain shedding.Immunoblot (IB) depicting the effect of BACE1 or γ-secretase on the generation of VEGFR1-CTF and VEGFR1-ICD.Presence of full-length VEGFR1_GFP_, VEGFR1_GFP_-CTF and VEGFR1_GFP_-ICD in membrane and cytosolic fractions determined by either using antibodies against the C-terminal of VEGFR1 or GFP.

Since we have previously reported that PEDF promotes γ-secretase-dependent cleavage of VEGFR1 (Cai et al, 2006, 2011b) and BACE is critical for the ectodomain cleavage of numerous transmembrane proteins (Klaver et al, 2010; Vassar et al, 2009) we hypothesized that BACE-dependent ectodomain shedding will, in addition to VEGFR1-NTF, generate a membrane-anchored C-terminal (VEGFR1-C-terminal fragment (CTF)) that is subsequently cleaved by γ-secretase, thus releasing an intracellular domain (VEGFR1-ICD) into the cytosol (Fig 3A). A GFP-tagged WT human VEGFR1 was stably expressed in a porcine aortic endothelial cell (PAEC) line devoid of VEGF receptors (Cai et al, 2011b). Total VEGFR1 levels remained similar for all treatments but were reduced in the presence of cycloheximide (Fig 3B). Exposure of cells to VEGF and PEDF in combination results in the shedding of an extracellular VEGFR1 fragment (VEGFR1-NTF), which could be blocked by inhibition of BACE1 but not γ-secretase inhibition (Fig 3C). The culture media also contains an ∼80 kDa sVEGFR1 (a soluble truncated splice variant of VEGFR1 that is usually secreted (Wu et al, 2010)) which is not affected by either PEDF exposure or BACE inhibition (Fig 3C). Treatment of cells with cycloheximide to inhibit protein synthesis blocks the accumulation of sVEGFR1 in the medium but has no significant effect on VEGFR1-NTC (Fig 3C). Taken together, these results demonstrate that in the presence of VEGF, PEDF induces BACE-mediated ectodomain shedding of VEGFR1 into the extracellular milieu. We next asked if BACE cleavage of the ectodomain of VEGFR1 is an essential prerequisite for transmembrane cleavage of VEGFR1 by γ-secretase. Cells treated with VEGF + PEDF showed a high intensity band for VEGFR1-ICD which is absent in both BACE inhibitor and γ-secretase inhibitor treated cells (Fig 3D). However, γ-secretase inhibition leads to an accumulation of uncleaved ∼115-kDa VEGFR1-CTF (Fig 3D). As expected, treatment by BACE inhibitor results in the absence of any fragments (Fig 3D). We next confirmed the subcellular location of these fragments via Western blot analysis of VEGFR1 for both membrane and cytosolic fractions using antibodies directed against the C-terminal domain of VEGFR1 and GFP, respectively (Fig 3E). A high intensity band for VEGFR1-ICD is visualized in the cytosol of cells treated with VEGF + PEDF while a low intensity band for VEGFR1-CTF is observed in the membrane. γ-secretase inhibition causes the disappearance of VEGFR1-ICD in the cytosolic fraction, accompanied by concomitant accumulation of VEGFR1-CTF in the membrane fraction. BACE inhibition results in the disappearance of the VEGFR1-ICD band from the cytosol; however, no accumulation of VEGFR1-CTF is observed in the membrane fraction (Fig 3E). Results consistent with these findings were obtained with western blot analysis using an antibody directed against GFP tag (Fig 3E).

**Figure 4:**
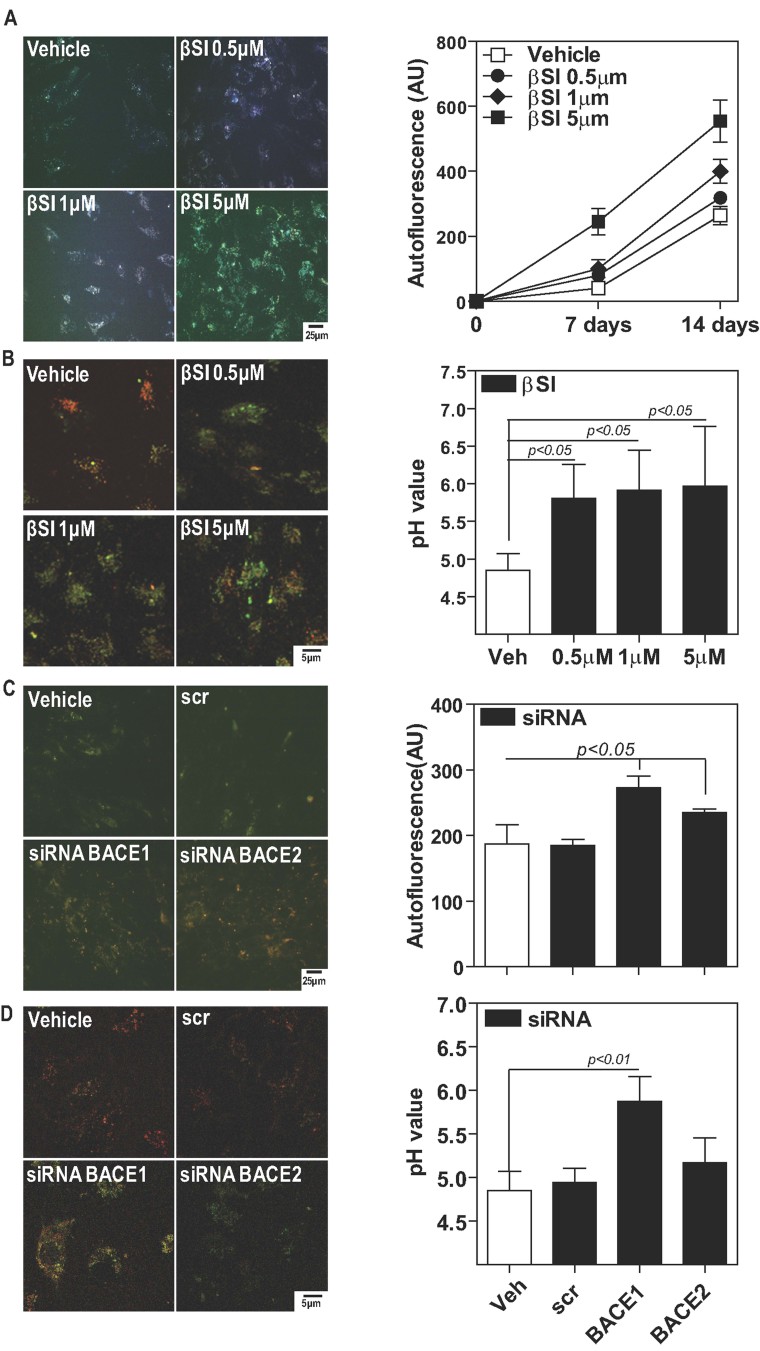
BACE1 inhibition enhances accumulation of lipofusion, decreases activity of cathepsin D and elevates lysosomal pH in cultured RPE cells Representative fluorescence micrographs and quantitation of autofluorescence by FACS (mean ± SEM, *n* = 6) in ARPE19 showed a dose-dependent increase in lipofusion granules in ARPE19 cells treated with BACE1 inhibitor for up to 14 days.Representative confocal microscopy images showing intralysosomal pH changes following exposure of ARPE19 cells to different concentrations of BACE1 inhibitor. pH was monitored using lysosensor yellow/blue dextran where a shift from red to green indicates a decrease in pH. The pH values were calculated using the emission intensity ratio at 535/450 nm with excitation at 340 nm for the lysosensor demonstrated increased lysosomal pH upon BACE 1 inhibition. The data are the mean ± SEM of values from three independent experiments.Representative fluorescence micrographs and quantitation of autofluorescence by FACS (mean ± SEM, *n* = 6) in ARPE19 showed a dose-dependent increase in lipofusion granules in ARPE19 cells treated with siRNA against BACE1 or BACE2 for up to 14 days. Vehicle only cells (Veh) and scrambled siRNA acted as the control.Representative confocal microscopy images showing intralysosomal pH changes following exposure of ARPE19 cells treated with siRNA against BACE1 or BACE2. Untreated cells and scrambled siRNA acted as the control. pH was monitored using lysosensor yellow/blue dextran where a shift from red to green indicates a decrease in pH. The pH values were calculated using the emission intensity ratio at 535/450 nm with excitation at 340 nm for the lysosensor and demonstrated increased lysosomal pH upon BACE 1 inhibition. The data are the mean ± SEM of values from three independent experiments.

To understand the role of BACE in lipofuscin accumulation we exposed cultured primary human RPE cells to BACE1 inhibitor or BACE1 small interfering RNA (siRNA). BACE1 inhibitor causes a time-dependent increase in autofluorescent granules over a 14-day period compared to vehicle only control and this is dose-dependent with the greatest accumulation (>250% over control) observed at the highest concentration of inhibitor used (Fig 4A). No overt cytotoxicity is observed at any of the time points. BACE1 inhibition causes a >40% reduction in the activity of Cathepsin D (Supporting Information Fig S5A), a lysosomal enzyme highly expressed in the RPE and implicated in lipofuscin formation (Rakoczy et al, 1996). Furthermore, BACE1 inhibition causes a significant increase in lysosomal pH compared to untreated control providing additional support for a link between BACE and lysosomal function (Fig 4B). However, to rule out the potential off target effect of the inhibitor we also treated cells with BACE1 siRNA and similarly observed an increase in both cellular autofluorescence and lysosomal pH (Fig 4C and D). BACE1 siRNA given by intravitreal injection also caused an increase in lipofuscin accumulation in a proportion of mouse RPE (Supporting Information Fig S5B). BACE2 knockdown had no effect on lysosomal pH but did induce a modest increase in lipofuscin.

## DISCUSSION

We demonstrate that both BACE1 and BACE2 are expressed in the normal mouse retina with BACE1 most highly expressed in the neural retina and BACE2 predominantly in the RPE/choroid. Not surprisingly knockout or inhibition of BACE leads to marked retinal pathology affecting the neural retina, the retinal vasculature and the RPE, and the phenotype differs between BACE1 and BACE2. These observations have major implications for the development and use of BACE inhibitors for AD as they could promote retinal damage and visual loss as a significant side effect. Indeed, May et al (2011) reported that a recently developed BACE1 inhibitor caused cytoplasmic accumulations of lipofuscin-like material in the retina, RPE enlargement and photoreceptor degeneration. The authors suggested that this was an off target effect but our studies in knockout mice and knockdown in adult animals would suggest different (May et al, 2011). May et al (2011) also reported that the eyes of BACE1^−/−^ mice were histologically unremarkable but did not undertake a detailed comparison with normal untreated eyes as we have done in this study.

The mechanism(s) by which BACE1 inhibition leads to retinal pathology and why this is prominent in the retinal vasculature and RPE is unclear. BACE1 is expressed in a variety of vascular and non-vascular retinal cell types and we are only now beginning to realize the range of BACE substrates (Klaver et al, 2010; Vassar et al, 2009; Woo et al, 2011). However, we have identified two potential mechanisms through which BACE1 inhibition can affect the retina by dysregulation of the VEGF signalling pathway and decreasing lysosomal efficiency.

We show that VEGFR1 ectodomain cleavage is a prerequisite for subsequent transmembrane cleavage by γ-secretase. Since this non-canonical pathway plays a critical role in the capacity of VEGFR1 to negatively regulate the pro-angiogenic role of VEGFR2 (Boulton et al, 2008a, b) its blockade by inhibition of BACE1 promotes angiogenesis in adult mice. The apparent contradiction between the inhibitory role of BACE in vascular development and stimulatory function in adult angiogenesis is not unexpected as the growth factor profile and cellular influences are very different. For example, VEGFR1 can mediate both pro- and anti-angiogenic outcomes dependent upon temporal and spatial expression of ligands as well as local microenvironment (Boulton et al, 2008b; Rahimi 2006). Furthermore, ectodomain shedding of the N-terminal fragment of VEGFR1 has the potential to neutralize extracellular VEGF and augment the role of sVEGFR1 (Rahimi et al, 2009).

The mechanism by which BACE elicits lysosomal changes is not known but BACE is known to be associated with lysosomes and the endosomal/lysosomal system is important in amyloid-beta production (Pasternak et al, 2004). Since, BACE inhibitors have variable activity toward cathepsin D (Bjorklund et al, 2010; Sealy et al, 2009) and the IC50 of BACE1 inhibitor (IV) used in this study is 15 nM for BACE1 and >50 µM for BACE2 and cathepsin D (Stachel et al, 2004), it is not likely that the inhibitor regulated lipofuscin accumulation via cathepsin D inhibition. We confirmed this using siRNA knockdown of BACE1 which supports a direct role for BACE in the endosomal/lysosomal system.

In conclusion, our observations raise potential concerns regarding the efficacy and safety of BACE inhibitors for the treatment of AD and emphasize that individuals treated with BACE inhibitors should be monitored carefully with regular ocular examinations. Up to 30% of those over 70 years of age experience age-related macular degeneration (AMD) and exhibit aberrant neovascularization and lipofuscin accumulation which will make these individuals particularly vulnerable to any adverse effects of BACE inhibition used for AD management.

## MATERIALS AND METHODS

### Animals and eye fixation and processing

BACE1^−/−^, BACE2^−/−^ and BACE1^−/−^ BACE2^−/−^ knockout mice and their WT controls (Dominguez et al, 2005) were used in these studies in accordance with the guidelines of the Institutional Animal Care and Use Committee at the Universities involved. BACE1^−/−^, BACE2^−/−^ and BACE1^−/−^BACE2^−/−^ mice ranging in age from 4 to 12 months were perfusion fixed in 4% paraformaldehyde and the posterior eyecup was processed and embedded in paraffin for routine histology and immunohistochemistry. For electron microscopy, another group of eyes were processed further by immersion fixation in 2.0% glutaraldehyde, postfixed in 1% osmium tetroxide in 0.1 M sodium cacodylate–HCl buffer (pH 7.4) with 7% sucrose at 4°C and embedded in LR-White resin. For retinal flat mounts animals were perfused through the left ventricle with 5 ml FITC-conjugated dextran (50 mg/ml) (FITC-Dextran, 2 × 106 MW) in 4% buffered paraformaldehyde (pH 7.4).

### Routine histology and transmission electron microscopy

Four micron wax embedded sections were stained with haematoxylin and eosin. A total of five sections per eye from four animals per group were analyzed by a masked observer. Digital photographs were taken between 2 and 4 mm temporal to the optic nerve head. Electron microscopy was performed as previously described (Qi et al, 2007a, b). The thickness of the neural retina and Bruch's membrane was determined morphometrically from 10 measurements from 10 micrographs per group using Image Pro software (Sengupta et al, 2009).

### Assessment and quantitation of vascular density

Retinal tissues were stained with agglutinin–FITC to visualize the blood vessels and anti-VEGFR1 antibody. The retinal vessel vascular area was measured in five random areas of five sections per eye and data presented as retinal vessel area in µm^2^ per µm^2^. In addition, average area of individual superficial retinal vessels was determined. Vascular changes were confirmed in retinas from FITC-dextran perfused animals by preparing retinal flat mounts on coverslips and assessing vascular structure in the superficial and deep retinal plexus by confocal microscopy.

### Quantification of apoptosis

Apoptosis was determined using an *in situ* cell death detection kit according to the manufacturer's instructions (Roche, Indianapolis, IN).

### Quantitation of lipofuscin

Confocal microscroscopy was used to measure autofluorescence in the RPE layer of knockout and WT eyes. Excitation frequency was 387 nm and emission frequency was 440–684 nm. Fluorescence intensity was assessed using Image J program available at http://rsbweb.nih.gov/.

### Funduscopic examination

For fundus imaging a Spectralis HRA + OCT (Heidelberg Engineering, Heidelberg, Germany) were used according to the instructions of the manufacturer. A 25 dp lens was used to adjust for the mouse eyes.

### Electroretinography (ERG)

ERGs were obtained as previously described (Brunner et al, 2010) with minor modifications. In brief, mice dark-adapted overnight received a flash series consisting of 10 steps started at −4.0 log cd s/m^2^ and reached 0.48 log cd s/m^2^. Oscillatory potentials were obtained with flash Intensity 0.48 log cd s/m^2^ immediately after the scotopic ERG. Stimulus duration was 250 ms. Stimulus (green light) energy were 0.48, 0.95 and 1.25 log cd s/m^2^ with bandpass filtering from 0.1 to 30 Hz. For a-wave recording, three additional flash energies were applied 0.97; 1.48 and 1.97 log cd s/m^2^. After recording the a-wave, the photopic ERG was recorded (0.7, 1.3 log cd s/m^2^; average of 10 recordings at 1.5 Hz). Subsequently, the animals were further light adapted for 10 min, and the photopic ERG was recorded again using flash energies from 0.7 to 1.3 log cd s/m^2^). The b-wave amplitude was determined from a-wave trough to b-wave peak, behind the last prominent oscillatory potential.

### Immunohistochemistry

Eyes from 6-week-old WT female C57BL/6J mice, BACE1^−/−^, BACE2^−/−^ and BACE1^−/−^ BACE2^−/−^ knockout mice and normal human eyes from 49- and 78-year-old male donors were processed for standard Paraffin embedding. Deparaffinized sections were treated with rodent deblocker (Biocare Medical, LIC) for antigen retrieval and then blocked with 10% normal goat sera, plus 5% BSA for 1 h in room temperature. Rabbit monoclonal anti-BACE1 (Cell Signaling, Danvers, MA) and anti-BACE2 (Santa Cruz Biotechnology, Inc, Santa Cruz, CA), were diluted 1:250 and 1:200, respectively, in PBS with 1% normal goat sera plus 1% BSA and incubated overnight at 4°C. Sections received a secondary antibody conjugated with Cy3 for 1 h at room temperature in the dark. Sections were covered with Vectashield mounting medium/DAPI (VECTOR LAB, Inc.) and images obtained using a Zeiss Fluorescence microscope.

### Real-time PCR

Eyes were enucleated from 6-week-old WT female C57BL/6J mice and the neuronal retina and RPE/choroidal-RPE were surgically separated. In addition mouse brains were isolated. Total RNA was extracted with Trizol total RNA isolation reagent (Invitrogen, Carlsbad, CA) and quantified using a Nanodrop Spectrophotometer. cDNA was synthesized from 1 µg total RNA from each sample using the iScript cDNA Synthesis Kit (BioRad, Hercules, CA). cDNA at 1:10 dilution was used for qRT-PCR on a Biorad C1000 Thermal Cycler. mRNA expression was assessed using the following BACE1 and BACE2 primer pairs: BACE1 forward primer: 5′-TCCGGCGGGAGTGGTATTATGAA-3′ and reverse primer 5′-ATCCGGGAACTTCTCCGTCGA-3′; BACE2 forward primers:5′-ATGGCTTCTGGACAGGGGCC-3′ and reverse primer 5′-ATCCGGAAGGAGCGACTGGC-3′. Their expression was normalized to the housekeeping gene, glyceraldehyde 3-phosphate dehydrogenase (GAPDH): forward primer 5′-CCCAGCAAGGACACTGAGCAAGAG-3′ and reverse primer 5′-CTAGGCCCCTCCTGTTATTATGGGG-3′. Relative transcript abundance was determined by using the ΔΔCt or ΔCt method after normalization with *GAPDH*. All samples were run in triplicate. Error bars represent standard error of the mean (SEM).

### Cell culture

Bovine retinal microvascular endothelial cells were isolated and cultured in endothelial cell basal medium with growth supplement (Invitrogen, Carlsbad, CA) as previously described (Cai et al, 2006). The cells were used within three passages. The human RPE cell line, ARPE 19 (ATCC #CRL-2302) were grown to confluence as previously described (Jarrett & Boulton, 2005) and then maintained in basal medium (Ham's F10 + 2% foetal bovine serum) for 7 days prior to experimentation.

### Generation of an endothelial cell line stably expressing VEGFR1

Porcine aortic endothelial cells with stable expression of VEGR1-GFP were prepared as previously described (Cai et al, 2011b).

### Growth factor and BACE inhibitor treatments

Endothelial cell cultures were rendered quiescent for 45 min in serum-free basal medium VEGF-A and PEDF (alone or in combination) were added at 100 ng/ml based on our previous studies (Cai et al, 2006, 2011a). Experiments were undertaken in the presence or absence of 0.5–5 µM BACE1 inhibitor IV (Sigma), 1 nM γ-secretase inhibitor N-[(3,5-difluorophenyl)acetyl]-L-alanyl-2-phenyl]glycine-1,1-dimethylethyl ester (DAPT) (Sigma) or BACE1 siRNA.

### Soluble VEGFR1 assay

To distinguish native sVEGFR1 from BACE ectodomain shed VEGFR1, the supernatant of microvascular endothelial cells was collected, concentrated with a Microcon filter (Millipore, Billerica, MT) and immunoprecipitated with an antibody against the N-terminal of VEGFR1 (Santa Cruz Biotechnology, Santa Cruz, CA). The resultant immunoprecipitates (IPs) were subjected to western blot analysis using antibodies against human native sVEGFR1 (RELLA Tech GmbH, Wolfenbűttel, Germany) and the N-terminal of VEGFR1 (Santa Cruz Biotechnology, Santa Cruz, CA).

### Subcellular protein extraction

Membrane and cytosolic proteins were purified from endothelial cells using the ProteoExtract™ subcellular proteome extraction kit (EMD Chemicals, Gibbstown, NJ). This kit preserves the integrity of the subcellular structures before and during extraction to prevent any mixing of the different subcellular compartments.

### Immunoprecipitation and western blotting

Immunoprecipitation and western blotting were performed as previously described (Cai et al, 2006, 2011a, c). In brief, cells were lysed in RIPA buffer containing protease inhibitors including lactacystin. Total proteins or proteins of subcellular fractions were IP with relevant antibody and separated by protein A/G-agarose, electrophoresed and transferred onto a nitrocellulose membrane. After blocking non-specific binding with 10% skimmed milk, the membranes were incubated overnight with the primary antibodies followed by horseradish peroxidase-conjugated secondary antibodies.

### *In vitro* angiogenesis assays

*In vitro* angiogenic activity of endothelial cells was analyzed quantitatively using proliferation, migration, and tubule formation models, as previously described (Cai et al, 2006). *Tubule formation*: endothelial cells were pretreated with growth factors for 24h prior to plating between two layers of 12.5% (v/v) Matrigel™ (BD Biosciences, Bedford, MA). Twenty-four hours later the tubule length (mm/mm^2^) was quantified from five random fields per well. *Proliferation*: the relative cell number was determined by crystal violet staining monitored at 540 nm. *Migration Assay (Wound Healing)*: confluent microvascular endothelial cells were pretreated with 25 mg/ml 5-fluorouracil for 5 min to prevent cell proliferation at the wound edge followed by 24 h treatment with growth factors (in the presence of 5-fluorouracil). Cell monolayers were wounded by a 1-cm^3^ tip pipette along one direction. Cell migration was monitored and photographed at initial wounding and at time points indicated under a phase microscope (4×). The images were quantified using Image J program and calculated as the distance (µm) migrated into the wound area.

### Laser-induced CNV model

The laser procedure was undertaken as previously described (Caballero et al, 2009; Shaw et al, 2006). Briefly, an argon green ophthalmic laser, set to deliver a 50 ms pulse at 200 mW with a 50 µm spot size, was used to rupture Bruch's membrane in three quadrants of the right eye. The left eye served as an untreated control. Animals received intravitreal injection at the time of laser and 7 days later with either saline or PEDF in the presence or absence of BACE1 inhibitor or BACE1 siRNA (1 µg/eye). Mice were sacrificed 14 days after laser injury. To determine vascular lesion volume a vascular specific dye, *Ricinus communis* Agglutinin I conjugated to rhodamine, was used to label whole flatmounts of RPE/choroid/sclera. Digital images were captured by using imaging software-SlideBook in a three-dimensional stacked manner to facilitate volumetric analysis from experimental and control samples with identical photomultiplier tube gain settings. The confocal images were then processed identically in experimental and control eyes and measured by using ImageJ software.

**The paper explained**

PROBLEM:

β-Secretase (BACE1) is responsible for the extracellular cleavage of amyloid precursor protein (APP), and BACE1 inhibitors are being actively developed to prevent amyloid-β accumulation in Alzheimer's disease (AD). Although BACE1 inhibitors provide a promising therapeutic target for AD, little is known about the effect of these agents on other neural tissues such as the retina.

RESULTS:

β-Secretase knockout mice develop significant retinal pathology including retinal thinning, vascular abnormalities and an increase in the age pigment, lipofuscin. BACE1-inhibition in adult WT mice increases both CNV and lipofuscin formation. BACE1 facilitates ectodomain shedding of VEGFR1 and is associated with lysosomal dysregulation.

IMPACT:

Since we have shown that BACE1 plays a key role in maintaining the retina, it is critically important to monitor carefully the potential ocular side effects of BACE inhibitors being developed to combat AD.

### Analysis of lipofuscin accumulation in cultured RPE cells

Lipofuscin levels in cultured cells were quantified by both FACS and image analysis at time 0 and weekly thereafter as previously described (Boulton et al, 1989; Wassell et al, 1998). The mean autofluorescence per 10,000 RPE cells was determined by the Image J program. Fluorescence microscopy was used to confirm lipofuscin accumulation.

### Measurement of lysosomal pH and cathepsin D activity

RPE cells were plated in 8-well coverglass bottom chambers (Lab-Tek, Naperville, IL) and cells were incubated in 1 mg/ml of the pH indicator LysoSensor Yellow/Blue dextran (Molecular Probes, Eugene, OR) for 12 h in the presence or absence of varying BACE inhibitor concentrations. The labelled cells were observed with a laser scanning confocal microscope using excitation at 360 nm and an emission filter at 450 nm and a long pass emission filter at 515 nm. Higher 530/450 nm ratios (*i.e.* a shift to green) correlate with a lower pH. Cathepsin D activity was measured in cell lysates using a fluorometric cathepsin D activity assay kit (Abcam, Cambridge, MA) and values are presented as the relative fluorescence units per million cells.

### Statistical analysis

All experiments were repeated at least three times. The results are expressed as the means ± SEM. Proliferation, migration, tube formation, FACs analysis, retinal thickness and vascular density were analyzed using a Student's *t*-test plus ANOVA with Bonferroni correction for multiple comparisons. The Mann–Whitney test was used to determine statistical significance of the laser densitometry data from western blots. *p* < 0.05 was considered statistically significant.

## Supplementary material

Detailed facts of importance to specialist readers are published as ”Supporting Information”. Such documents are peer-reviewed, but not copy-edited or typeset. They are made available as submitted by the authors.
