# Supplementary material for: β-Secretase (BACE1) inhibition causes retinal pathology by vascular dysregulation and accumulation of age pigment
Source: EMBO Mol Med. 2012 Aug 20;4(9):980–91. doi: 10.1002/emmm.201101084 (PMC3491829; doi:10.1002/emmm.201101084)
Supplement: Supplementary file 1 [file emmm0004-0980-SD1.pdf]

EMBO
Molecular Medicine

Manuscript EMM-2011-01084

β-SECRETASE (BACE1) INHIBITION CAUSES RETINAL PATHOLOGY BY VASCULAR DYSREGULATION AND ACCUMULATION OF AGE PIGMENT

Jun Cai, Xiaoping Qi, Norbert Kociok, Sergej Skosyrski, Alonso Emilio, Qing Ruan, Song Han, Li Liu, Zhijuan Chen, Catherine Bowes Rickman, Todd Golde, Maria B Grant, Paul Saftig, Lutgarde Serneels, Bart de Strooper, Antonia M. Joussen, Michael E Boulton

Corresponding author: Michael Boulton, University of Florida

Review timeline:

Transfer date:	18 November 2011
Editorial Decision:	09 January 2012
Appeal:	23 January 2012
Editorial Decision:	23 December 2011
Revision received:	11 May 2012
Editorial Decision:	18 June 2012
Revision received:	23 June 2012
Accepted:	28 June 2012

Transaction Report:

(Note: With the exception of the correction of typographical or spelling errors that could be a source of ambiguity, letters and reports are not edited. The original formatting of letters and referee reports may not be reflected in this compilation.)

1st Editorial Decision 09 January 2012

Thank you for the submission of your research manuscript to our editorial office and please accept my apologies for not replying earlier due to an accumulated delay that occurred during the holidays. We have now heard back from the three referees whom we asked to evaluate your manuscript.

As you will see from the enclosed reports, all three reviewers find your study of potential medical interest. However, while referee #1 is rather positive, referees #2 and #3 are not and raise serious concerns regarding the conclusiveness of the data, pinpointing major methodological issues that preclude a solid interpretation of the experimental evidence provided. The reviewers call for a considerable amount of additional experimentation to resolve these issues and do not feel that this would be addressable in a timely fashion to be considered further in our Journal.

In light of these criticisms, the amount of work likely to be required to address them, and the fact that, EMBO Molecular Medicine can only invite revision of papers that receive enthusiastic support from a majority of referees, I am afraid that we do not feel it would be productive to call for a revised version of your manuscript at this stage and therefore we cannot offer to publish it.

Given the potential interest of the findings, we would, however, have no objection to consider a new manuscript on the same topic if at some time in the near future you obtained data that would considerably strengthen the message of the study and address the referees concerns in full. To be completely clear, however, I would like to stress that if you were to send a new manuscript this

would be treated as a new submission rather than a revision and would be reviewed afresh, in particular with respect to the literature and the novelty of your findings at the time of resubmission. If you decide to follow this route, please make sure nevertheless to upload a letter of response to the referees' comments.

At this stage of analysis, though, I am sorry to have to disappoint you. I nevertheless hope, that the referee comments will be helpful in your continued work in this area and I thank you for considering EMBO Molecular Medicine.

Yours sincerely,

Editor
EMBO Molecular Medicine

***** Reviewer's comments *****

Referee #1:

The manuscript from Cai et al. provides a rigorous perspective on b-secretase function in mouse retinal biology and as a specific effector of retinal vascular architecture. The authors have experience working with aspartyl proteases with an earlier study published on gamma-secretase pro-angiogenic and vasopermeability function via PEDF/VEGF signaling. The main findings of the work are that b-secretase deficient animals (BACE1/2) have an accelerated aging vascular/neuronal and RPE phenotype. In postnatal biology, this signaling pathway appears to regulate VEGFR1 cleavage and functions in a pro-angiogenic fashion. Even though the pathway appears rather complicated, the authors do a commendable job of simplifying this and elegantly testing their hypotheses. The experiments are well-designed and executed, and the manuscript/figures are presented in a professional and logical layout. There are a few minor concerns which should be addressed prior to publication:

1. Pharmacologic inhibition of b-secretase was performed showing increased laser-induced CNV in the mouse model. The authors should confirm this data with additional laser injury studies in the BACE1, BACE 2 and BACE1/2 deficient mice.

2. Please provide fundus/FA images in a supplemental figure for BACE deficient mice.

3. Has this phenotype been confirmed in the commercially available Jax mice, and why did the authors choose this particular transgenic?

4. In the description of the developmental phenotype, to better understand the effects on retinal architecture and function, ERG data is strongly recommended.

5. I noticed several typos throughout the manuscript.

Referee #2:

The manuscript is divided into three parts. In the first part, the authors describe a retinal pathology observed in mice deficient for beta-secretase (BACE1) and/or its relative BACE2. The pathology in BACE1 KO mice included retinal thinning, apoptosis, reduced vascular density, and increased accumulation of lipofuscin. The second part of the manuscript adresses the role of BACE1 in the modulation of VEGF-A stimulated endothelial responses and the processing of VEGFR-1 in vitro. The corresponding results show that inhibition of VEGF-A mediated endothelial responses by PEDF is dependent on BACE1 activity, as is the processing of a membrane-anchored C-terminal fragment of VEGFR1 into an intracellular fragment. In the third part of the manuscript, it is shown that BACE1 inhibition in cultured RPE cells leads to increased lipofuscin accumulation. The authors conclude that the retinal pathology that develops in BACE1 deficient mice is due, in part, to dysregulation of the VEGFR1 signaling pathway and a decrease in lysosomal efficiency.

The results presented in the manuscript are well described and interesting. However, the study fails

to demonstrate that the retinal phenotype observed in BACE KO mice is indeed primarily caused by the described effects of BACE inhibition in vitro. In particular, it remains unclear to which extent BACE deficiency in endothelial cells and dysregulation of VEGFR1 contributes to the development of the observed retinal pathology. Can the authors exclude that BACE1 deficiency in non-endothelial cells causes retinal pathology? In the absence of such evidence, the major conclusion drawn by the authors remains speculative. Endothelial cell-specific ablation of BACE1 could be helpful in this respect.

Additional points of criticism:

1.) The authors hypothesize on page 3 that "BACE knockout could accelerate retinal pathology" because "deposition of Abeta is associated with retinal degeneration". However, according to the arguments presented, BACE1 deficiency should result in reduced production of Abeta and, consequently, would be not be expected to accelerate retinal pathology.
2.) The authors point out that BACE2 shares 68 percent sequence identity with BACE1. Is there any evidence for functional redundancy between BACE1 and BACE2? Did the authors test whether BACE1 expression is up-regulated in BACE2-deficient mice?
3.) It appears that blood vessels are enlarged in BACE1 Ko mice (Figure 2a). This should be quantified.
4.) Electron microscopy of BACE Ko retinas indicates pericyte loss. This should be complemented by immunohistological detection of pericyte markers, such as NG2, or PDGFRbeta.
5.) It is unclear what "inhibitory role of BACE in vascular development" (page 5) refers to.
6.) Lysates used for IP in Figure 3 should be analysed for total VEGFR1 expression and tubulin. Was total VEGFR1 expression altered by any of the treatments?

Referee #3:

This manuscript describes the effect of BACE deficiency on retinal morphology using knockout animals and characterized in vitro one of the mechanisms of BACE dependent inhibition of PEDF activity. Using mostly immunochemistry and EM, the authors showed that BACE -/- animals display many retinal vascular anomalies and pathological accumulation of lipofuscin. Using in vitro cellular models, they demonstrated that BACE inhibition blocks the anti-angiogenic effects of PEDF and that this effect is due in part to the proteic release of VEGFR1 ectodomain.

BACE inhibition is one of the front-runner strategies for the treatment of AD, therefore the determination of BACE function in other neuronal tissue such as the retina is of high interest. In addition the demonstration that BACE is involved in the angiogenic activity of PEFD by cleaving the extra-cellular domain of VEGFR1 is novel and intriguing. However, this ms appears somewhat premature and suffers from numerous methodological flaws. Comments and suggestions to improve the quality of the manuscript are described bellow.

First and foremost, because this study is limited to older animals, it is impossible to know if any of the phenotypes described in the ko animals are developmental or due to progressive degeneration (as suggested by the authors). In addition most of the in vivo characterization suffers from major discrepancies between the anomalies described in the text and what is shown or quantified in the figure section. The examples are numerous:

- Page 3: "In BACE1-/- KO....... caused depopulation of the ganglion cell layer". A demonstration and quantification of apoptotic GC using TUNEL or caspase-3 marker is necessary. Extensive cell apoptosis and retinal thinning depends on the assumption that retinal development is normal in the KO animals, which again remains to be demonstrated.

- Page 3: "RPE atrophy and a marked increase in the age pigment lipofuscin are observed (Fig. 1C)". There is no evidence of RPE atrophy in the images provided. Legend of Figure 1 contains multiple similar claims. Example page 14: " Electron micrographs of BACE1-/- RPE/choroid appeared to contain less melanin, increased lipofuscin and thinning in Bruch's membrane (BM) compared to WT. In addition, the choroid showed a decrease in choroidal capillaries in knockout mice". These statements are not supported by the TEM pictures provided. In addition the quality of the TEM is too poor to properly discern the Bruch's membrane layers or the choriocapillaris lumen.

- Changes in the retinal and choroidal vascular density described are improperly characterized. In figure 2 a and b, the quality of the vascular staining is particularly poor making this analysis questionable. Proper techniques such as retina flat-mount preparation and staining of endothelial cells using specific markers such as CD31, Lectins and/or Collagen IV for the endothelium basal membrane should be employed. The "capillary" shown in figure 2a is obviously a large artery and nuclei are observed while it is described as an acellular capillary (this higher magnification appears to belong to the anti-BACE2 staining picture provide in supplemental figure 2b (??)). In addition, none of the figures provided show the "severe choroidal vasculature attenuation" stated page 4. Quantification of choroidal vascular density requires the use of specific methods such as vascular corrosion cast or lectin perfusion. Simply observation of histological sections or TEM is insufficient (page 4 and suppl. Fig 1).

Contrary to the authors claim page 7 the dose of BACE inhibitor used in this study (5µM) is high enough to be able to induce off-target inhibition as the IC50 of beta-secretase inhibitor IV for BACE-2 and cathepsin D are 0.23µM and 7.6µM receptively (IC50 described by the reference provided and confirmed by the inhibitor manufacturer). Therefore some of the in vitro observations on EC angiogenesis and lipofuscin accumulation could be due to cathepsin D inhibition.

The concentrations of VEGF and PEDF used in the in vitro study are extremely high and outside the clinically relevant range. In addition, the authors described previously that maximal HREC proliferation and migration can be obtained with a VEGF concentration of 5ng/ml. It is unclear why VEGFR1 cleavage requires the presence of both VEGF and PEDF.

Finally, the authors failed to correlate their in vivo analysis of the BACE KO animals to their in vitro study on VEGFR1 cleavage. It is unclear if the N-ter portion of VEGFR1 released by BACE is able to bind VEGF and act as an endogenous VEGF inhibitor similar to soluble VEGFR1. No evidence of cleavage of VEGFR1 (or lack of it in the KO animals) or alteration in VEGFR1 signaling in vivo is provided, therefore the mechanism for deleterious effects of BACE inhibition in vivo remains undetermined.

Other comments:

Text:
- Page 3, second paragraph: The justification that BACE ko could accelerate retinal pathology based on the facts that BACE1 is expressed in the retina and that Amyloid beta deposition is associated with retinal degeneration is obviously highly counter-intuitive. This paragraph should be corrected.

Figure section:
- As a wide range of animal age has been used (6 to 12 months), the age of the KO and WT control animals needs to be provided for each figure.
- Supplemental Figure 3: pictures a and b are not at the same magnification.

Appeal 23 January 2012

We thank you for your response regarding our manuscript EMM-2011-01084-T entitled " -secretase inhibition causes retinal vascular pathology and accumulation of age pigment". We are clearly disappointed with your decision to reject this paper and are writing to ask you to reconsider your decision based on the following information which confirms this as a high impact and cutting edge piece of research which is of major interest to a wide audience. We apologize if we did not make this sufficiently clear in the original manuscript.

1. Given the clinical impact of our research in highlighting the severe retinal phenotype associated with BACE1 inhibition, taken together with the increasing numbers of BACE1 inhibitors going into clinical trials for Alzheimer's disease, we should stress the importance to have these data published as soon as possible. This is emphasized by the recent press release of Eli Lilly in which they have stopped their BACE1 phase 2 clinical trials due to ocular complications but based this on an off

target of their drug rather than a direct mechanism-based effect. Our data indicate a much more fundamental problem than what is thought by Eli Lilly and other Pharma in this area and indicate that ALL BACE inhibitors must be tested carefully for this ocular side effect. Furthermore, this work was enthusiastically received at the recent Keystone angiogenesis meeting with participants eager for a publication.

2. Overall the three referees agree with the phenotype and that it is an important finding. We have extensive histological data we can include which can address the concerns of the reviewers and which we omitted in our first submission due to concerns over space limitations. Furthermore, we now have data for BACE1 siRNA knockdown in vivo and in vivo which recapitulates our data using the pharmacological BACE inhibitor thus confirming that this cannot be an off target effect. We would add this data to any revised submission.

3. Both our in vitro and vivo data emphasize the important role that BACE1 plays in retinal pathophysiology, even if they are mechanistically not completely worked out. We perhaps should have discussed the mechanistic aspects not as definitive conclusions but more in terms of potential pathways that could contribute to BACE effects in the retina. It may actually take a long time to determine the exact mechanism(s) by which BACE affects retinal function as it is expressed in a variety of vascular and non-vascular retinal cell types and we are only just beginning to become aware of BACE substrates. A case example is -secretase which was originally identified for its transmembrane cleavage of APP. It is now known to have over 50 substrates, many of which contribute to retinal pathophysiology but the precise pathways remain elusive.

4. We are easily able to address all the comments and suggestions made by reviewers 1 and 2 and have already undertaken many of the additional experiments. However, with respect to reviewer 2 we would like to comment that we are not attempting to make an association between Abeta and AMD and that Dr Bowes-Rickman who is the forerunner of Abeta research in the retina, and a co-author on this paper, does not see this study as a contradiction but rather supportive. Also, we did demonstrate that BACE1 expression is not up-regulated in BACE2-deficient mice in the supplementary data.

5. We see the biggest problem as reviewer 3 who although making some pertinent comments has misunderstood some of the data presented and not fully realized the timely and clinical impact of our research. First, the aim of this paper is to emphasize that BACE1 plays an important role in retinal physiology and that it's knockdown whether in transgenic or adult animals results in retinal pathology. Our new siRNA studies clearly confirm that this is a progressive degeneration. Second, we have now undertaken the additional staining and can confirm the presence of apoptotic GC using caspase-3 staining. Third, the discrepancies between the anomalies reported by the reviewer largely represent a misunderstanding of what we presented, in part due to ambiguity on our part. As in descriptive studies we showed representative photomicrographs. However, we have an extensive archive of figures which clearly confirm all that is reported or quantified in the figure section and we will be happy to include these in the supplementary data section. Fourth, we have collectively over 60 years of experience in visualizing retinal vessels via a range of techniques and strongly disagree with the reviewer 3's comment that retina flat-mount preparation and staining of endothelial cells using specific markers should be employed. Retinal flat mounts gives insufficient detail and only provides a 2D structure of blood vessels and lose the ability to understand the 3-D structure of the vasculature and how if interfaces with the remaining retina. Furthermore, we did use an endothelial-specific lectin for our staining. However, we do agree that better labeling of the figures may have deflected some of this reviewers criticisms including the perception that "severe choroidal vasculature attenuation" was referring to BACE1 (which did not show any significant vascular changes) when we were describing BACE2. Fifth, any off target effect of the BACE inhibitor is now excluded with our new BACE siRNA data. Sixth, the concentrations of VEGF and PEDF used in the in vitro studies have been widely used by us and others and the reason that VEGFR1 cleavage requires the presence of both VEGF and PEDF has been extensively described in previous publications which were referenced in this manuscript. Finally, as discussed above, both our in vitro and vivo data emphasize the important role that BACE1 plays in retinal pathophysiology, even if they are mechanistically not completely worked out. We perhaps should have discussed the mechanistic aspects not as definitive conclusions but more in terms of potential pathways that could contribute to BACE effects in the retina.

We hope that we have managed to convince you of the broad interest and the major impact of our study and hope that we can persuade you to reconsider your decision. Should you reconsider, we can provide a revised paper within 4-6 weeks.

We thank you for taking the time to read this email.

2nd Editorial Decision 23 December 2011

Thank you for your letter asking us to reconsider our decision. I have read it with interest and discuss once more your manuscript and the letter with my colleagues.

First let me say that we do appreciate the high medical interest of your findings and agree that, in light of the stopped clinical trial from Eli Lilly, your data should indeed be reported.

We also greatly appreciate that you have done some siRNA experiments *in vivo* targeting BACE1. Providing that these new data are robust and conclusive, they may address the tissue specificity issue reported by both referees #2 and #3.

Therefore, given the data you are ready to provide and understanding that all issues raised by all referees should be convincingly addressed, we would like to invite a resubmission of your manuscript.

At this stage, I would like to draw your attention to our journal formatting requirements and would encourage you to modify your manuscript adequately. Please see our guide to authors for more details:
http://onlinelibrary.wiley.com/journal/10.1002/%28ISSN%291757-4684/homepage/ForAuthors.html

Revised manuscripts should be submitted within three months of a request for revision; they will otherwise be treated as new submissions, except under exceptional circumstances in which a short extension is obtained from the editor. Also, the length of the revised manuscript may not exceed 60,000 characters (including spaces) and, including figures, the paper must ultimately fit onto optimally ten pages of the journal. You may consider including any peripheral data (but not methods in their entirety) in the form of Supplementary information.

I look forward to seeing a revised form of your manuscript as soon as possible.

Yours sincerely,

Editor
EMBO Molecular Medicine

1st Revision - authors' response 11 May 2012

RESPONSE TO REVIEWERS

We thank the reviewers for their helpful comments. We have now addressed these concerns and the manuscript is greatly strengthened.

Response to reviewer 1

1. Pharmacologic inhibition of β-secretase was performed showing increased laser-induced CNV in the mouse model. The authors should confirm this data with additional laser injury studies in the BACE1, BACE 2 and BACE1/2 deficient mice.

Due to the histological changes in BACE1-/- eyes it was deemed inappropriate to undertake the CNV model in these animals as differences in vascular lesion formation may simply reflect differences in tissue structure rather than a direct effect of BACE. To address this we used BACE1-/- siRNA to transiently knockdown BACE1 and were able to show that this increased CNV lesion volume. This is now included in Figure 2h.

2. Please provide fundus/FA images in a supplemental figure for BACE deficient mice.

We now provide fundus images from these animals in supplemental figure 1.

3. Has this phenotype been confirmed in the commercially available Jax mice, and why did the authors choose this particular transgenic?

The targeted Bace1 and Bace2 null mice were generated in our institute and characterized extensively in Dominguez et al, 2005). The Bace1 strain used in this study was made independently but is equivalent to the strain004714 available from Jax mice. Both strains were made by insertion of selection markers disturbing the BACE1 ORF resulting in complete loss of the Bace1 protein The Bace 2 KO mice which were generated in our institute, have been made available as Jax mice (stock code 005618).

4. In the description of the developmental phenotype, to better understand the effects on retinal architecture and function, ERG data is strongly recommended.

We now supply ERG data in Supplementary figure 1. There was a decrease in photopic ERG but not scotopic. However, these were 3 month old animals and it may well be that at 18 months a greater decline in ERG would be observed.

5. I noticed several typos throughout the manuscript.

We have corrected the typos.

<u>Response to reviewer 2</u>

1. The study fails to demonstrate that the retinal phenotype observed in BACE KO mice is indeed primarily caused by the described effects of BACE inhibition in vitro. In particular, it remains unclear to which extent BACE deficiency in endothelial cells and dysregulation of VEGFR1 contributes to the development of the observed retinal pathology. Can the authors exclude that BACE1 deficiency in non-endothelial cells causes retinal pathology? In the absence of such evidence, the major conclusion drawn by the authors remains speculative. Endothelial cell-specific ablation of BACE1 could be helpful in this respect.

Both our in vitro and vivo data emphasize the important roles that BACE1 plays in retinal pathophysiology, even if they are mechanistically not completely worked out. We perhaps should have discussed the mechanistic aspects not as definitive conclusions but more in terms of potential pathways that could contribute to BACE effects in the retina. It may actually take a long time to determine the exact mechanism(s) by which BACE affects retinal function as it is expressed in a variety of vascular and non-vascular retinal cell types and we are only just beginning to become aware of BACE substrates. A case example is γ-secretase which was originally identified for its transmembrane cleavage of APP. It is now known to have over 50 substrates, many of which contribute to retinal pathophysiology but the precise pathways remains unknown. We have now modified the manuscript accordingly to reflect that we have identified two potential pathways.

2. The authors hypothesize on page 3 that "BACE knockout could accelerate retinal pathology" because "deposition of Abeta is associated with retinal degeneration". However, according to the

arguments presented, BACE1 deficiency should result in reduced production of Abeta and, consequently, would not be expected to accelerate retinal pathology.

We do not consider this a contradiction and have reworded the text accordingly. It is evident from our data (see Fig 1) that BACE1 is strongly expressed even in the normal wild type retina of young mice and thus would be expected to perform an important physiological function. As has been shown by one of the co-authors (Bowes Rickman) there is minimal Abeta expression in the young retina. However, Abeta levels are noticeable elevated in older animals, and those in humans with AMD and this may reflect either an increase in substrate or BACE1 itself.

3. The authors point out that BACE2 shares 68 percent sequence identity with BACE1. Is there any evidence for functional redundancy between BACE1 and BACE2? Did the authors test whether BACE1 expression is up-regulated in BACE2-deficient mice?

We show expression levels for BACE1 and BACE2 in WT, BACE1-/- and BACE2-/- mice in Figure 1 and supplementary Figure 3. As expected, staining for BACE1 and BACE2 is absent from their respective knockouts while the complementary BACE is expressed at levels consistent with wild type controls but not at increased compensatory levels. This is now made clear in the results.

4. It appears that blood vessels are enlarged in BACE1 Ko mice (Figure 2a). This should be quantified.

The reviewer is correct that the superficial retinal vessels are enlarged. This is now shown in Figure 2e.

5. Electron microscopy of BACE KO retinas indicates pericyte loss. This should be complemented by immunohistological detection of pericyte markers, such as NG2, or PDGFRbeta.

This is now confirmed in Figure 2c

6. It is unclear what "inhibitory role of BACE in vascular development" (page 5) refers to.

This sentence has now been clarified.

7. Lysates used for IP in Figure 3 should be analysed for total VEGFR1 expression and tubulin. Was total VEGFR1 expression altered by any of the treatments?

There is no major change in total VEGFR1. This is now shown in Figure 3b

<u>Response to reviewer 3</u>

1. First and foremost, because this study is limited to older animals, it is impossible to know if any of the phenotypes described in the KO animals are developmental or due to progressive degeneration (as suggested by the authors).

The aim of this study is to highlighting the severe retinal phenotype associated with BACE1 inhibition. This is of major importance given the increasing numbers of BACE1 inhibitors going into clinical trials for Alzheimer's disease. Our observations raise potential concerns regarding the efficacy and safety of BACE inhibitors for the treatment of Alzheimer's disease (AD) and emphasize that individuals treated with BACE inhibitors should be monitored carefully with regular ocular examinations. Up to 30% of those over 70 years of age experience AMD and exhibit aberrant neovascularization and lipofuscin accumulation which will make these individuals particularly vulnerable to any adverse effects of BACE inhibition used for AD management. Furthermore, claims that this could be an off target effect is not substantiated by our BACE1 siRNA studies. It was not our intention in this paper to undertake an in depth analysis of the role of BACE1 in retinal development or to study disease progression. We have modified the text accordingly.

2. "In BACE1-/- KO....... caused depopulation of the ganglion cell layer". A demonstration and quantification of apoptotic GC using TUNEL or caspase-3 marker is necessary. Extensive cell apoptosis and retinal thinning depends on the assumption that retinal development is normal in the KO animals, which again remains to be demonstrated.

We have now provided additional evidence for apoptosis using TUNEL staining and undertaken quantification (Supplementary Fig 1c and Figure 1d).

3. "RPE atrophy and a marked increase in the age pigment lipofuscin are observed (Fig. 1C)". There is no evidence of RPE atrophy in the images provided. Legend of Figure 1 contains multiple similar claims. For example page 14: " Electron micrographs of BACE1-/- RPE/choroid appeared to contain less melanin, increased lipofuscin and thinning in Bruch's membrane (BM) compared to WT. In addition, the choroid showed a decrease in choroidal capillaries in knockout mice". These statements are not supported by the TEM pictures provided. In addition the quality of the TEM is too poor to properly discern the Bruch's membrane layers or the choriocapillaris lumen.

We apologize to the reviewer for any ambiguity our images and text may have caused. We have added a new figure which clearly shows atrophy (i.e. thinning) of the RPE layer in BACE1-/- animals when compared to WT (Figure 1f). We have improved the resolution of the images and removed reference to melanin granules and changes in choroidal capillaries in BACE1-/- mice. We have also revised the text to better reflect our findings.

4. Changes in the retinal and choroidal vascular density described are improperly characterized. In figure 2 a and b, the quality of the vascular staining is particularly poor making this analysis questionable. Proper techniques such as retina flat-mount preparation and staining of endothelial cells using specific markers such as CD31, Lectins and/or Collagen IV for the endothelium basal membrane should be employed. The "capillary" shown in figure 2a is obviously a large artery and nuclei are observed while it is described as an acellular capillary (this higher magnification appears to belong to the anti-BACE2 staining picture provide in supplemental figure 2b (??)). In addition, none of the figures provided show the "severe choroidal vasculature attenuation" stated page 4. Quantification of choroidal vascular density requires the use of specific methods such as vascular corrosion cast or lectin perfusion. Simply observation of histological sections or TEM is insufficient (page 4 and suppl. Fig 1).

Once again, we apologize to the reviewer for any ambiguity our images and text may have caused. We replaced Figure 2a to better reflect the retinal vascular changes in cross section. We have now added retinal flat mounts (Figure 2b) which support our findings in figure 2a. We have added supplemental figure 4a which emphasizes the lack of endothelial cells lining capillaries. We have removed reference to choroidal vascular attenuation. The only choroidal changes observed were in the BACE2-/- mice and this has now been corrected in the manuscript. There were no noticeable choroidal changes in BACE1-/- mice.

5. Contrary to the authors claim page 7 the dose of BACE inhibitor used in this study (5µM) is high enough to be able to induce off-target inhibition as the IC50 of beta-secretase inhibitor IV for BACE-2 and cathepsin D are 0.23µM and 7.6µM receptively (IC50 described by the reference provided and confirmed by the inhibitor manufacturer). Therefore some of the in vitro observations on EC angiogenesis and lipofuscin accumulation could be due to cathepsin D inhibition.

We agree, and to address these concerns we repeated the experiments using BACE1 siRNA. We obtained very similar results so feel confident that this is not an off target effect. This additional data has been incorporated into the manuscript.

6. The concentrations of VEGF and PEDF used in the in vitro study are extremely high and outside the clinically relevant range. In addition, the authors described previously that maximal HREC proliferation and migration can be obtained with a VEGF concentration of 5ng/ml. It is unclear why VEGFR1 cleavage requires the presence of both VEGF and PEDF.

This has been extensively addressed in our previous publications (Cai et al, 2006 *J Biol Chem*; Cai et al, 2006 *Microvascular Res*; Cai et al, *PLoS ONE* 2011; Cai et al, *J Biol Chem* 2011; Qi et al, *Invest Ophthalmol Vis Sci* 2012), most of which are referenced in this manuscript. In brief, VEGF and PEDF concentrations are optimal for the cultured porcine and bovine retinal microvascular endothelial cells used in these studies and have been widely used by us and others. Although PEDF alone is sufficient to activate secretases, cleavage of VEGFR1 can only occur after binding VEGF. Ligand binding is required to cause a conformational change in the receptor and open up secretase cleavage sites.

7. Finally, the authors failed to correlate their in vivo analysis of the BACE KO animals to their in vitro study on VEGFR1 cleavage. It is unclear if the N-ter portion of VEGFR1 released by BACE is able to bind VEGF and act as an endogenous VEGF inhibitor similar to soluble VEGFR1. No evidence of cleavage of VEGFR1 (or lack of it in the KO animals) or alteration in VEGFR1 signalling in vivo is provided, therefore the mechanism for deleterious effects of BACE inhibition in vivo remains undetermined.

Both our in vitro and vivo data emphasize the important roles that BACE1 plays in retinal pathophysiology, even if they are mechanistically not completely worked out. We perhaps should have discussed the mechanistic aspects not as definitive conclusions but more in terms of potential pathways that could contribute to BACE effects in the retina. It may actually take a long time to determine the exact mechanism(s) by which BACE affects retinal function as it is expressed in a variety of vascular and non-vascular retinal cell types and we are only just beginning to become aware of BACE substrates. A case example is γ-secretase which was originally identified for its transmembrane cleavage of APP. It is now known to have over 50 substrates, many of which contribute to retinal pathophysiology but the precise pathways remain elusive. We have now modified the manuscript accordingly to reflect that we have identified two potential pathways by which BACE inhibition could result in retinal pathology.

We did not investigate whether the N-ter portion of VEGFR1 released by BACE is able to bind VEGF and act as an endogenous VEGF inhibitor as we have previously reported that the N-ter portion of VEGFR1 "is a functional protein and that it inhibits VEGF-mediated proliferation" (Rahimi et al, *Cancer Res* 2009).

8. Page 3, second paragraph: The justification that BACE ko could accelerate retinal pathology based on the facts that BACE1 is expressed in the retina and that Amyloid beta deposition is associated with retinal degeneration is obviously highly counter-intuitive. This paragraph should be corrected.

We do not consider this a contradiction. It is evident from our data (see Fig 1) that BACE1 is strongly expressed even in the normal wild type retina of young mice and thus would be expected to perform an important physiological function. As has been shown by one of the co-authors (Bowes Rickman) there is minimal Abeta expression in the young retina. However, Abeta levels accumulate in older animals, and those with AMD and this may reflect either an increase in substrate or BACE1 itself. In fact, immunotherapy targeting Abeta in an AMD mouse model protects it from RPE damage and loss of visual function.

9. As a wide range of animal age has been used (3 to 12 months), the age of the KO and WT control animals needs to be provided for each figure.

This is now added.

10. Supplemental Figure 3: pictures a and b are not at the same magnification.

This has now been addressed and is new supplemental figure 2.

© EMBO

2nd Editorial Decision 18 June 2012

Thank you for the submission of your revised manuscript to EMBO Molecular Medicine. We have now received the enclosed reports from the referees that were asked to re-assess it. As you will see the reviewers are now supportive and I am pleased to inform you that we will be able to accept your manuscript pending the following final amendments:

As you can see, Referee #3 still has minor comments that should be addressed in the text as appropriate and in an enclosed point-by-point response letter.

On a more editorial note:
- Please reformat your article in EMBO Molecular Medicine format including references. See our guidelines for help:
http://onlinelibrary.wiley.com/journal/10.1002/%28ISSN%291757-4684/homepage/ForAuthors.html#manuscript

- For Figures and Figure legends, please use capital letters (A, B, C...)

- Please provide up to 5 keywords only

- for The Paper Explained section, please provide a bit more background in the "Problem" chapter

- Regarding figures, please decrease the contrast that was set too high for all the western blots (figure 3 and Figure S4E), as the panels are white.

Please submit your revised manuscript within two weeks. I look forward to seeing a revised form of your article as soon as possible.

Yours sincerely,

Editor
EMBO Molecular Medicine

***** Reviewer's comments *****

Referee #2:

The authors have addressed important points of criticism raised by the reviewers. However, the mechanistical insight is still limited because it remains unclear whether the effects of BACE inhibition observed in vitro contribute to the development of the retinal pathology.

Referee #3:

In their revision, the authors have addressed the reviewers concerns and substantially improved their manuscript through the addition of critical data. They made significant improvements in their description of the neuronal and vascular phenotypic consequence of BACE1 and BACE2 genetic inactivation. Somewhat of a disconnect between the in vitro characterization of BACE function on PEDF dependant VEGFR1 release and the in vivo retinal phenotype (such as reduced vascular density) remains but the authors clearly identify these issues in the text. More importantly, the authors refined their in vivo analysis, added new data and control experiments as requested. In addition, the authors clearly demonstrated that BACE1 inhibition in RPE via reagents or siRNA technique in vitro recapitulates the increased autofluorescence observed in vitro. I still have some comments (see below) that should be addressed before publication.
The authors should explain the difference between photopic and scotopic ERG (for non specialist readers) and explain why the cone-only response was affected.
Supplemental figure 1c shows Tunel+ fluorescence in all cells (GCL and ONL) of the BACE1-/- retina. If it is real and not an artefact of preparation or imaging, a much significant retinal

degeneration should be expected.

Supplemental figure 1d: what are the changes (white dots and white shadow) described in the autofluorescence fundus? It is unclear how this relates to the retinal phenotype described in the main manuscript.
Could the authors comment of the localization of the atrophic RPE compared to the regions with autofluorescent RPE?

2nd Revision - Authors' Response 23 June 2012

RESPONSE TO REVIEWERS

We thank the reviewers for their highly supportive comments. We have now addressed the additional comments from reviewer 3 and have completed all the editorial issues.

Response to reviewer 3

1. The authors should explain the difference between photopic and scotopic ERG (for non specialist readers) and explain why the cone-only response was affected.

We have now clarified in the text that scotopic ERG is the rod photoreceptor response under low light conditions and photopic ERG is the cone photoreceptor response under well-lit conditions allowing color perception. We cannot explain why the cone-only response is affected but would anticipate that in older animals we may see a loss in both scotopic and photopic ERG.

2. Supplemental figure 1c shows Tunel+ fluorescence in all cells (GCL and ONL) of the BACE1-/- retina. If it is real and not an artefact of preparation or imaging, a much significant retinal degeneration should be expected.

- We have now provided a more representative photomicrograph for Supplemental fig 1c

3. Supplemental figure 1d: what are the changes (white dots and white shadow) described in the autofluorescence fundus? It is unclear how this relates to the retinal phenotype described in the main manuscript.

Autofluorescence fundus images of BACE1-/- mice exhibited a white “shadow” around the main vessels suggestive of inflammation while in BACE2-/- mice there were white dots concentrated at the optic nerve indicating focal areas of lipofuscin hyperfluorescence. This is now clarified in the main text.

4. Could the authors comment of the localization of the atrophic RPE compared to the regions with autofluorescent RPE?

- The areas of atrophy were always associated with elevated lipofuscin. This is now clarified in the text.
