# Supplementary material for: β-Secretase (BACE1) inhibition causes retinal pathology by vascular dysregulation and accumulation of age pigment
Source: EMBO Mol Med. 2012 Aug 20;4(9):980–91. doi: 10.1002/emmm.201101084 (PMC3491829; doi:10.1002/emmm.201101084)
Supplement: Supplementary file 2 [file emmm0004-0980-SD2.pdf]

Supplementary data for:

β-Secretase (BACE1) inhibition causes retinal pathology by vascular dysregulation and accumulation of age pigment

Jun Cai, Xiaoping Qi, Norbert Kociok, Sergej Skosyrski, Alonso Emilio, Qing Ruan, Song Han, Li Liu, Zhijuan Chen, Catherine Bowes Rickman, Todd Golde, Maria B Grant, Paul Saftig, Lutgarde Serneels, Bart de Strooper, Antonia M. Joussen, Michael E Boulton

Table of contents:

**Supplementary figure 1. Functional and structural changes in the BACE1-/- mouse retina compared to wild type control.**	**2**
**Supplementary figure 2. Morphological analysis of BACE2-/- and BACE1-/- BACE2-/- mice.**	**4**
**Supplementary figure 3. Expression of BACE in normal and BACE knockout retinas.**	**5**
**Supplementary figure 4. The effect of BACE inhibition on vascular pathology and retinal endothelial cell proliferation and migration plus BACE1 expression in retinal cells.**	**7**
**Supplementary figure 5. The effect of BACE1 inhibition on lipofuscin accumulation and Cathepsin D activity in the RPE.**	**9**

Suppl Figure 1

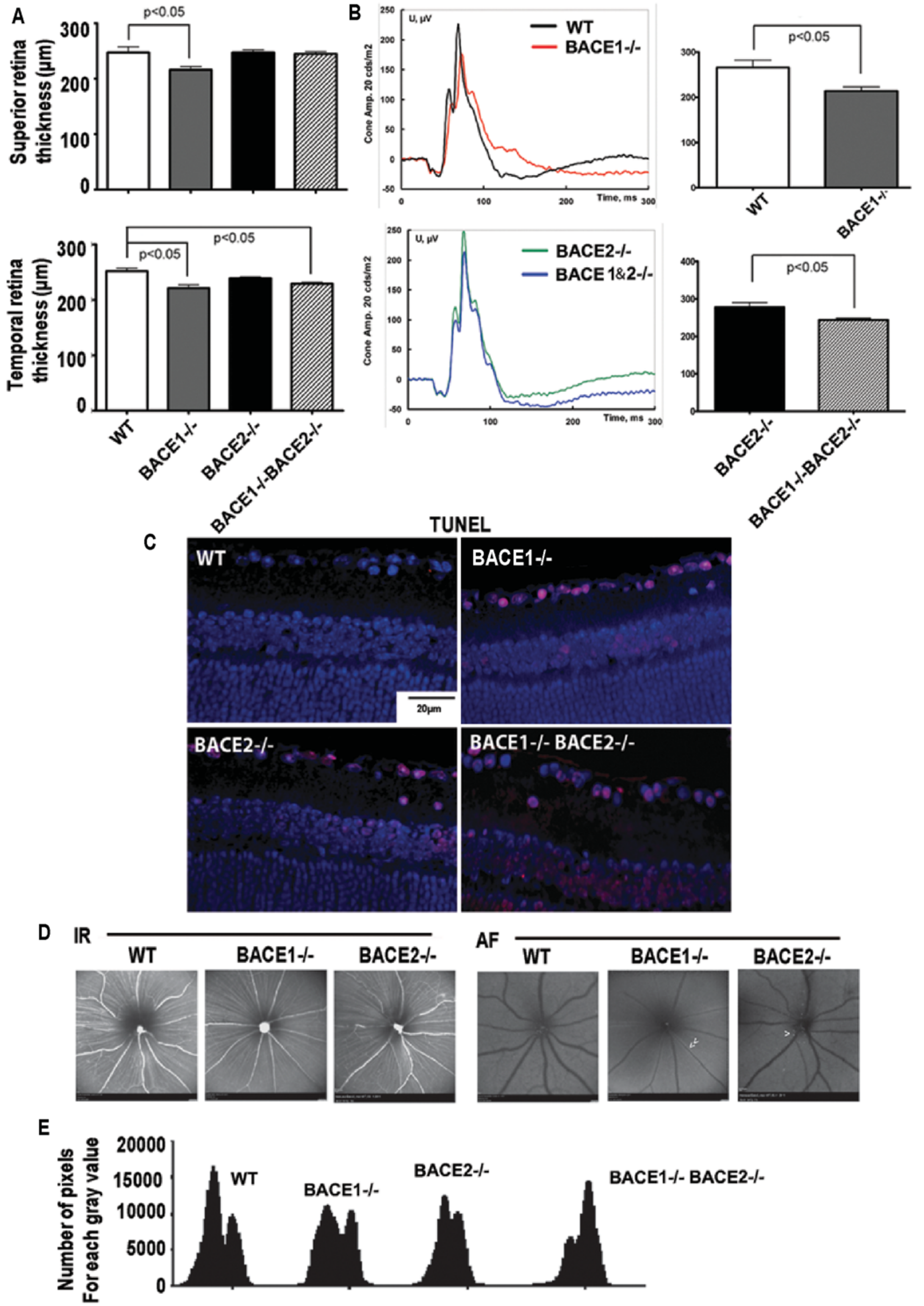

Supplementary figure 1. Functional and structural changes in the BACE1-/- mouse retina compared to wild type control. A - OCT measurement of the average retina thickness at the superior, and temporal retina. In all three regions the retina thickness is reduced in BACE1-/- mice when compared to WT mice. **B - P**hotopic ERG at 20cds/m^2 after 10min light adaption showing a significant decrease in amplitude for both BACE1-/- when compared to age-matched WT mice (3 months) and BACE1-/-/ BACE2-/- when compared to BACE2-/- mice. **C -** Apoptosis is greatly increased in 4 month old BACE1-/- and BACE1-/-/BACE1-/- mice when compared to age-matched WT. Sections show TUNEL positive cells (red) with nuclei labeled with DAPI (blue). **D -** Representative infrared images (IF) and autofluorescence images (AF) of mouse retinas. In BACE1-/- mice there is a white "shadow" (<<) around half of the main vessels. In BACE2-/- mice there are white dots (>) concentrated at the optic nerve. **E -** Histogram analysis of the AF pictures (taken at the same focus -4.25) in BACE1-/- and BACE1-/- BACE2-/- mice showing the autofluorescence is increased. The WT histogram has the main peak at darker gray values and a smaller peak at brighter values, whereas the BACE1-/- histogram has two similar peaks at the darker and brighter values. The BACE2-/- histogram is similar to the WT histogram and on the BACE1-/-BACE2-/- histogram the peak at the brighter gray values is much larger than the peak at the darker values.

Suppl Figure 2

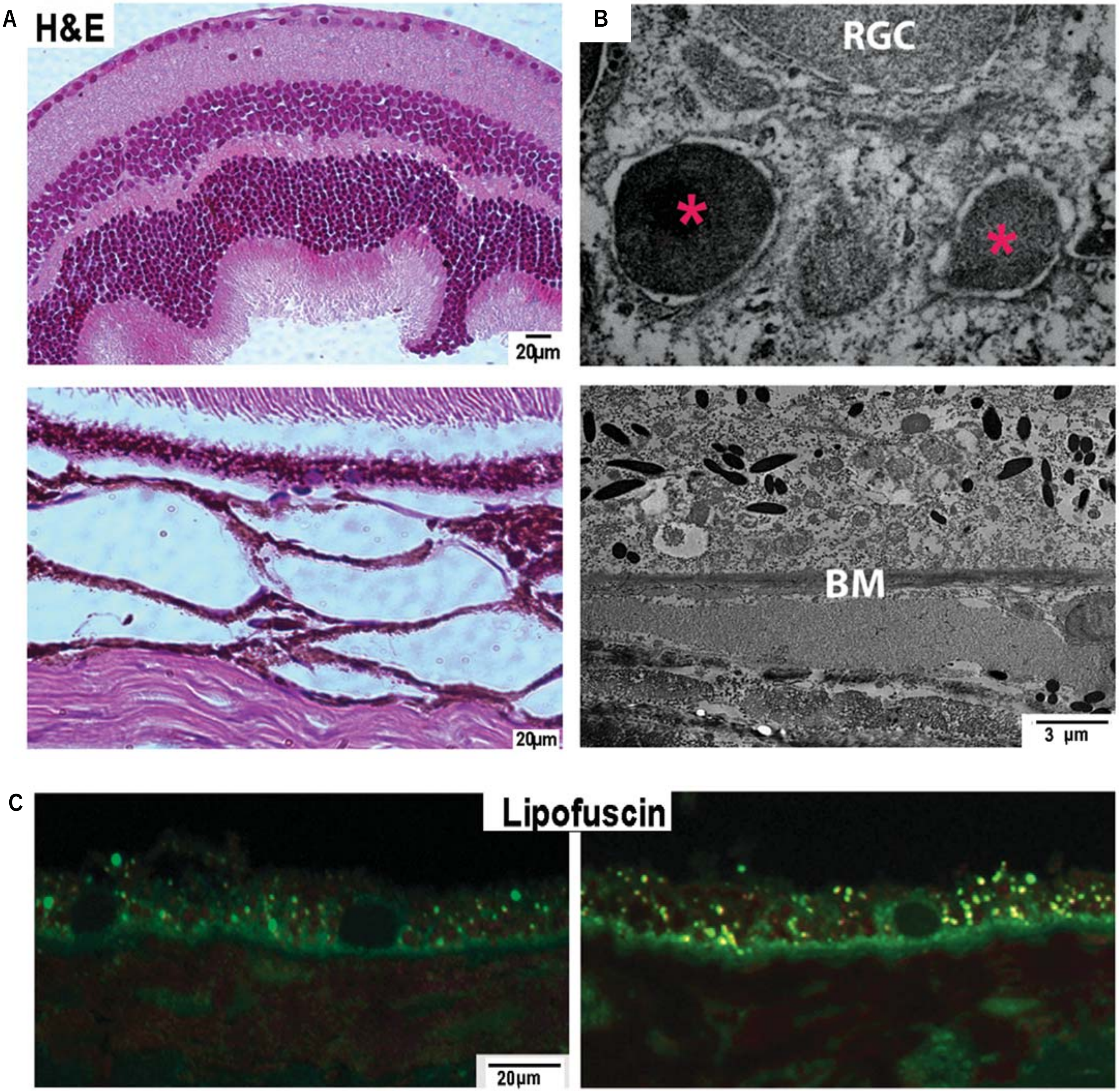

Supplementary figure 2. Morphological analysis of BACE2-/- and BACE1-/- BACE2-/- mice. Animals were 4 months and age-matched with WT. **A -** Representative hematoxylin/eosin staining images of retinas from BACE2-/- mice. In BACE2-/- mice the neural retina appeared relatively normal although occasional foci of neural retinal hyperplasia were observed and there were regions where the choroid was extensively disrupted. **B** – Electron microscopy of BACE1-/-BACE2-/- double knockout animals exhibited a retinal phenotype similar to that observed for BACE1-/- mice including RGC death and Bruch's membrane thickening. **C** – fluorescence images of lipofuscin accumulation in the overlying RPE was less in BACE2-/- mice than observed in BACE1-/- animals (Fig. 1) and BACE1-/- BACE2-/-.

Suppl Figure 3

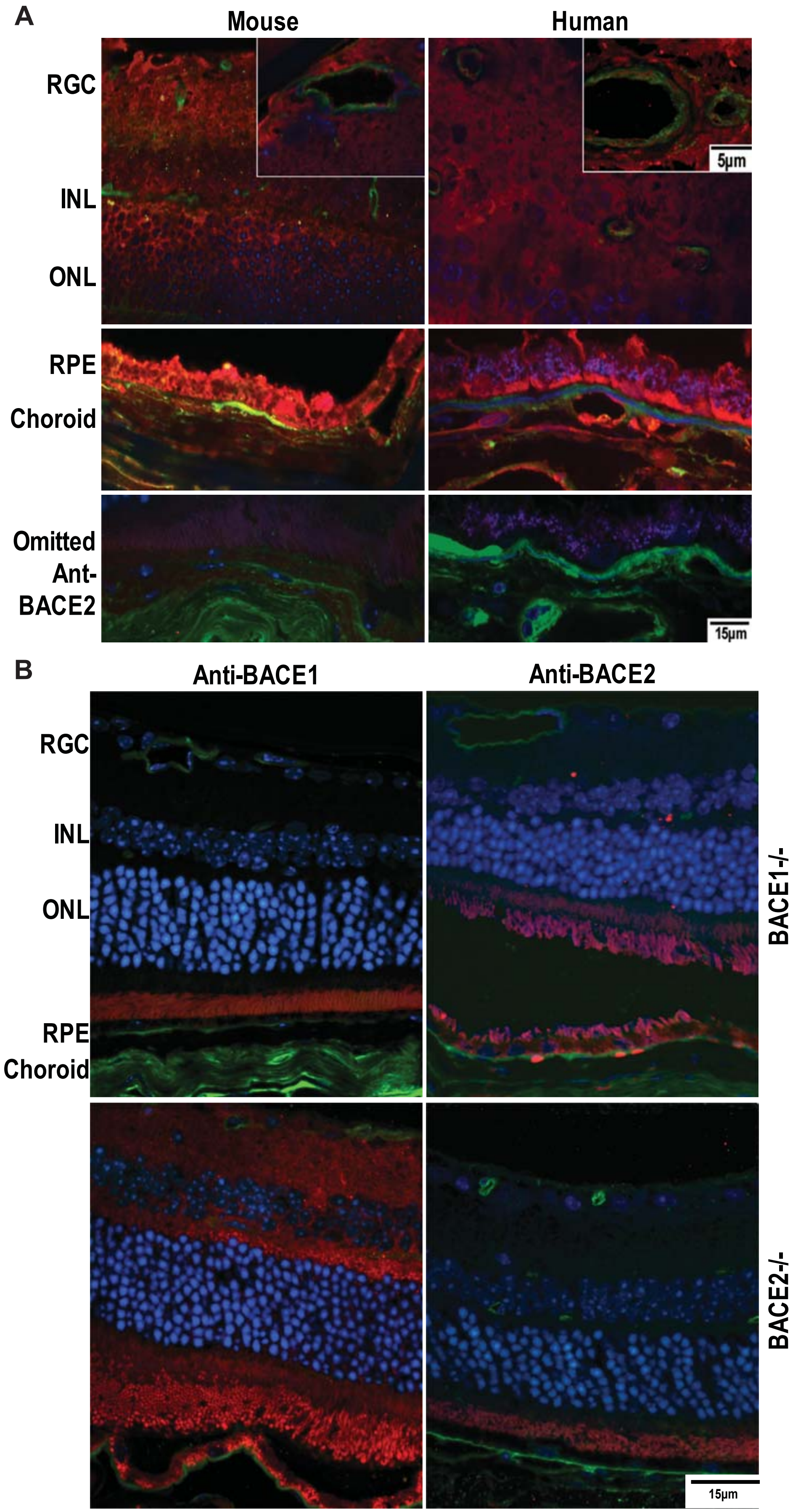

Supplementary figure 3. Expression of BACE in normal and BACE knockout retinas. A – Animals were 4 months and age-matched with WT. Representative figures showing detection of BACE2 expression in the retina of wild-type mouse and human eyes. Paraffin sections were immunostained using an antibody against BACE2 (red) and dual staining with agglutinin–FITC (green) to visualize the vasculature. BACE2 was strongly expressed in the RPE and choroid of both normal mouse and human eyes but was only weakly expressed in the retina. Inset shows higher magnification of retinal vessels. **B** – Immunostaining of BACE1-/- (red) and BACE2-/- (red) eyes with antibodies against BACE1 and BACE2 to confirm absence of expression of the relevant BACE isoform in the knockouts and the presence of the other isoform. Controls are omission of the primary antibody.

Suppl Figure 4

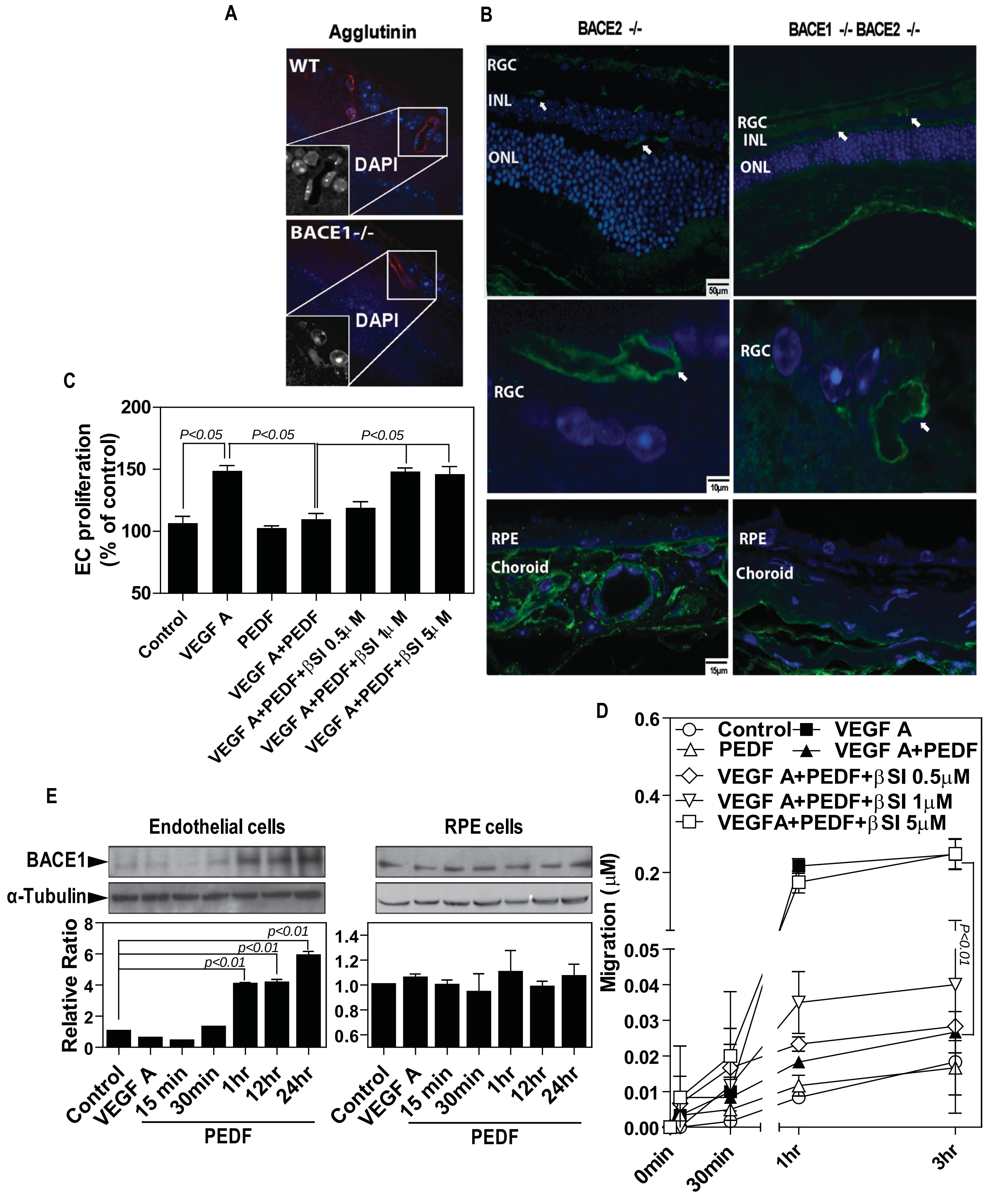

Supplementary figure 4. The effect of BACE inhibition on vascular pathology and retinal endothelial cell proliferation and migration plus BACE1 expression in retinal cells. A - Animals were 4 months and age-matched with WT. Dual staining with agglutinin to visualize retinal vascular endothelial cells (red) and DAPI (blue) showing an absence of nucli associated with vessels in the BACE1-/- mice when compared to WT. **B** - Representative retinal sections stained with agglutinin–FITC (green) to visualize the retinal vasculature. The vasculature density and integrity appeared near normal in BACE2-/- mice. However, BACE1-/- BACE2-/- animals showed decreased retinal capillaries and the presence of acellular capillaries similar to that observed for BACE1-/- mice. Representative choroidal sections of BACE2-/- and BACE1-/- BACE2-/- animals showed major changes in the appearance of choroidal vessels in the BACE2 -/- mice. **C-E** - Retinal microvascular endothelial cells were treated with VEGF (100ng/ml) and/or PEDF (100ng/ml), in the presence of or the absence of 0.5, 1 or 5 μM β-secretase inhibitor IV (β-SI) for varying times. Results are mean ± SEM of at least three independent experiments. **C** - Proliferation studies in which the relative cell numbers were determined by crystal violet staining. **D -** Cell migration was assessed by quantification of scratch wound closure (relative to 0h). To avoid cell proliferation at the wound edge the cell monolayers were treated with 25mg/ml 5-fluorouracil for 5 min before treatment with growth factors. **E** – Representative immunoblots and densitometric analysis (Mean±SEM, n=3 independent experiments) of BACE1 expression in lysates from microvascular endothelial cells and ARPE19 cells treated with VEGF (100ng/ml) in the presence or absence of PEDF (100ng/ml). α-tubulin was used as the loading control for normalization. The ratio relative to control is arbitrarily presented as 1.

Suppl Figure 5

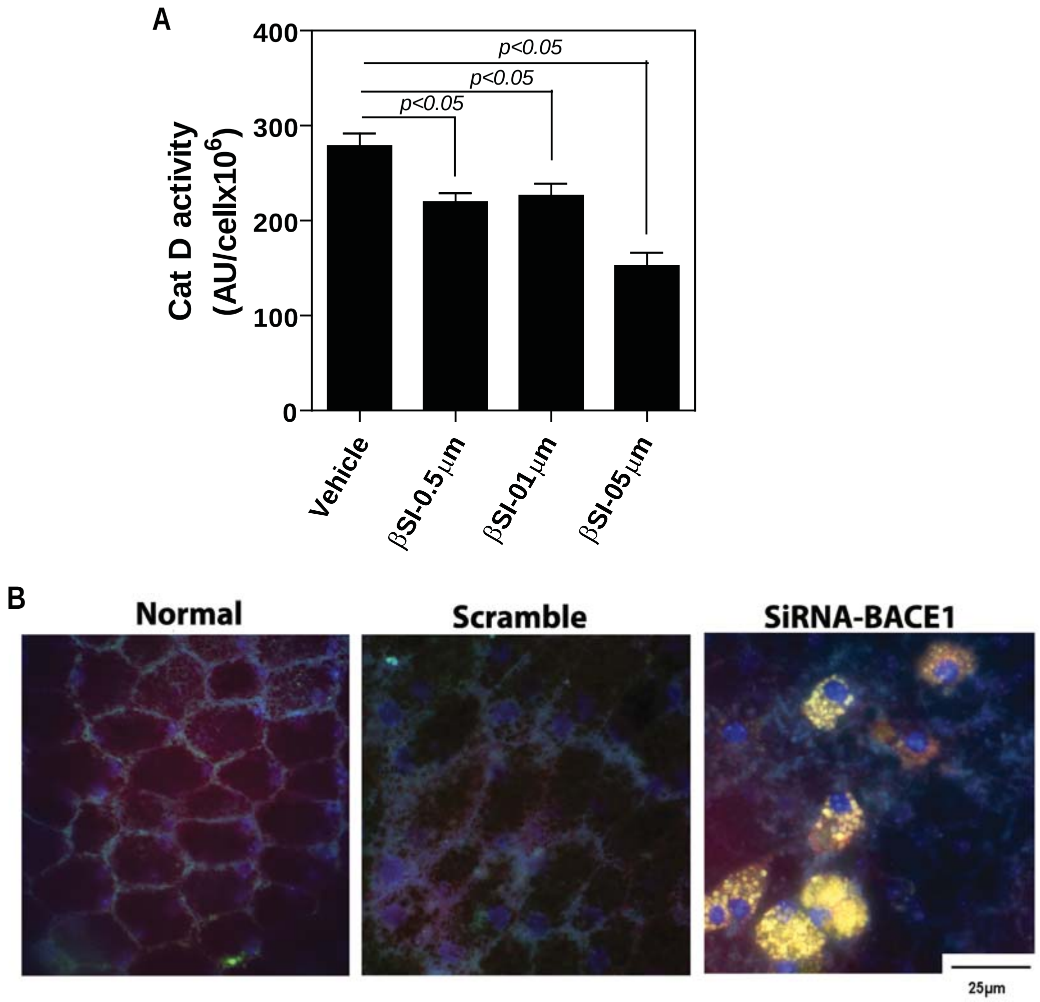

Supplementary figure 5. The effect of BACE1 inhibition on lipofuscin accumulation and Cathepsin D activity in the RPE. A – Cathepsin D activity in ARPE19 cells treated with different concentrations of BACE1 inhibitor determined by a fluorometric assay. The results were presented as the relative fluorescence units per million cells. (Mean±SEM, n=6). **B -** Confocal image of RPE flat mounts from eyes of 2 month old mice receiving intravitreal injection of either saline vehicle control (normal), scrambled siRNA or BACE1 siRNA.
